# Single-cell RNA sequencing reveals cell type-specific immune regulation associated with human neuromyelitis optica spectrum disorder

**DOI:** 10.3389/fimmu.2024.1322125

**Published:** 2024-02-19

**Authors:** Yushu Jiang, Shuhua Dai, Rui Pang, Lingzhi Qin, Milan Zhang, Huiqin Liu, Xiaojuan Wang, Jiewen Zhang, Gongxin Peng, Yongchao Wang, Wei Li

**Affiliations:** ^1^Department of Neurology, Henan Joint International Research Laboratory Of Accurate Diagnosis, Treatment, Research And Development, Henan Provincial People’s Hospital, People’s Hospital of Zhengzhou University, Zhengzhou, Henan, China; ^2^Department of Neurology, Zhoukou Central Hospital, Zhoukou, Henan, China; ^3^Center for Bioinformatics, Institute of Basic Medical Sciences, Chinese Academy of Medical Sciences & School of Basic Medicine, Peking Union Medical College, Beijing, China; ^4^Department of Neurology, People’s Hospital of Yexian, Pingdingshan, Henan, China

**Keywords:** neuromyelitis optica spectrum disorder, peripheral blood mononuclear cell, single-cell RNA sequencing, B cell, T cell, myeloid cell

## Abstract

**Introduction:**

One rare type of autoimmune disease is called neuromyelitis optica spectrum disorder (NMOSD) and the peripheral immune characteristics of NMOSD remain unclear.

**Methods:**

Here, single-cell RNA sequencing (scRNA-seq) is used to characterize peripheral blood mononuclear cells from individuals with NMOSD.

**Results:**

The differentiation and activation of lymphocytes, expansion of myeloid cells, and an excessive inflammatory response in innate immunity are observed. Flow cytometry analyses confirm a significant increase in the percentage of plasma cells among B cells in NMOSD. NMOSD patients exhibit an elevated percentage of CD8+ T cells within the T cell population. Oligoclonal expansions of B cell receptors are observed after therapy. Additionally, individuals with NMOSD exhibit elevated expression of CXCL8, IL7, IL18, TNFSF13, IFNG, and NLRP3.

**Discussion:**

Peripheral immune response high-dimensional single-cell profiling identifies immune cell subsets specific to a certain disease and identifies possible new targets for NMOSD.

## 1 Introduction

Optic neuritis (ON) and transverse myelitis (TM) recurrent episodes are characteristics of the rare autoimmune disease neuromyelitis optica spectrum disorder (NMOSD) (1). These episodes are associated with the existence of particular serum antibodies against the water channel aquaporin-4 (AQP4) (1, 2). NMOSD is more common among non-White populations, representing at least one third of cases of central nervous system inflammation (3). It predominantly affects females (4). While intravenous glucocorticoids are the preferred treatment option during the acute phase of NMOSD, only a small percentage of patients (19%) experience short-term symptom relief following treatment (5). Furthermore, it should be noted that serum AQP4-IgG levels do not always correspond directly to the severity of NMOSD. Although a serological AQP4-IgG test is necessary for the diagnosis of NMOSD, the titer level does not reflect the severity of neurological prognosis or ongoing disease activity (6). Therefore, further research is needed to identify additional markers and underlying mechanisms of NMOSD.

Cross-reactivity with bacterial antigens that resemble AQP4 or an antibody-mediated response to AQP4 expressed by tumor cells may be the initial mechanisms underlying NMOSD (1). Defects in immune checkpoints can lead to the emergence of autoreactive B cells, which are pivotal in the pathology of NMOSD (7). B cells in NMOSD patients, when stimulated by interleukin-6 (IL-6) with CD4+ T cells, can generate AQP4-IgG (8). The blood-brain barrier can be broken down by activated B and T cells, which can release pro-inflammatory cytokines. This allows AQP4-IgG and other immune cells, including as macrophages and granulocytes, to infiltrate the central nervous system (9). The binding of AQP4-IgG to AQP4 induces lytic and sublytic damage to astrocytes, which may vary in terms of reversibility (10, 11). Active demyelinating regions of NMOSD show increased infiltration by various immune subsets, including granulocytes, macrophages, eosinophils, lymphocytes, and plasma cells, compared to still-myelinated areas (12).

Previous studies on NMOSD have mainly relied on low-throughput assays for immunophenotyping, which have been limited to a small number of cell types and markers (7, 13). While the auto-antigen and effector mechanisms of NMOSD have been well characterized, the complexity and heterogeneity of the immune system cannot be fully captured using these methods (10). Therefore, high-throughput assays are needed to comprehensively understand the immune system regulatory patterns in NMOSD. Single-cell RNA sequencing (scRNA-seq) has offered a potent tool for deciphering the immune system in human diseases (14). By applying scRNA-seq to NMOSD research, it becomes possible to capture the transcriptomic profiles of individual immune cells at a high resolution, thereby enabling a more profound comprehension of the diverse immune cell populations and their functional states in the disease.

In this study, we conducted scRNA-seq analysis on peripheral blood mononuclear cells (PBMCs) from NMOSD patients. Initially, 20 major cell groups were identified and the primary alterations occurring in these cell types were evaluated. Moreover, involvement of plasma cells, memory B cells, and CD8+ T cells in NMOSD was observed, characterized by oligoclonal expansion of B-cell receptors (BCRs). Additionally, positive regulation of monocyte activation was observed in NMOSD following steroid therapy. Our data indicate that myeloid cells serve as the primary source of proinflammatory mediators and represent potential therapeutic targets in PBMCs. In conclusion, our findings help characterize the cellular pathological mechanisms and deepen our understanding of the immune system heterogeneity associated with NMOSD. This contributes to the development of innovative and effective therapeutic strategies.

## 2 Materials and methods

### 2.1 Processing of patient samples

This study received approval from the Ethics Committee of Henan Provincial People’s Hospital. A total of 53 suspected NMOSD patients were enrolled in the Neurology Department of Henan Provincial People’s Hospital from January 2019 to June 2023.

The following were the inclusion criteria for patients with NMOSD: (1) diagnosis of NMOSD based on the International consensus diagnostic criteria (15); (2) testing sero-positive for AQP4 antibody; (3) age between16–80 years. The following were the exclusion criteria for patients with NMOSD: (1) concurrent infectious diseases (e.g., meningitis, encephalitis or peripheral infection); (2) myelin oligodendrocyte glycoprotein antibody-associated disease (MOGAD) or multiple sclerosis (MS); (3) immunologically relevant comorbidities; (4) history of malignant tumors; (5) pregnancy; and (6) history of immunotherapy (e.g., steroid, immunoglobulin, plasma exchange, immunosuppressant, or monoclonal antibody treatments). Ultimately, 43 patients with NMOSD were included in the study cohort, with six patients undergoing scRNA-seq and flow cytometric validation, while the remaining 37 patients only underwent flow cytometric validation. Additionally, ten age-matched HCs were enrolled. The first cohort, consisting of six NMOSD patients and five HCs, was utilized for scRNA-seq. The second cohort, comprising 43 NMOSD patients and 10 HCs, was used for flow cytometric analysis (**Supplementary Table 1**). Among the 43 NMOSD patients, high-dose methylprednisolone pulse therapy was administered. We collected peripheral blood samples the day before and ten days after the initiation of steroid therapy. Before the second peripheral blood sample collection, they had not received monoclonal antibody therapy or any other immunosuppressive medications. All patients and HCs provided informed consent prior to participation.

### 2.2 Preparing and sequencing single cell samples, constructing single cell library

To begin the process, the majority red blood cells were removed from blood samples by dilution in phosphate-buffered saline (PBS), and the samples were converted into single cell suspensions using a human peripheral blood lymphocyte isolation solution. The PBMCs were then cryopreserved in liquid nitrogen and transported using dry ice to maintain their viability and molecular integrity. Upon thawing the frozen aliquots, 0.4% trypan blue staining selectively stains non-viable cells, enabling accurate determination of cell viability. A Countess^®^ II Automatic Cell Counter (Thermo Fisher Scientific) was used for quality inspection. Samples were considered qualified if the cell viability was greater than 80% and the cell concentration ranged from 700-1200 cells/μL. Next, the 5’ Library and Gel Bead Kit, V(D)J Enrichment Kit, and the Chromium Single Cell 5’ library preparation kit user guide provided by 10X Genomics were utilized. A mixture of 75μL Master Mix, cell suspension, 40μL gel beads containing barcode information, droplets of 280μL oil were introduced into several chambers of a Chromium Chip B. Through a microfluidic “double cross” system, oil droplets encapsulated gel beads, single cells, Master Mix, and oil, resulting in the formation of Gel Bead-In-Emulsions (GEMs). The GEMs were collected and combined, and the gel beads dissolved within oil droplets, releasing barcode primers. Meanwhile, cells underwent lysis and released RNA. Reverse transcription was initiated when mRNA, reverse transcriptase, poly dT reverse transcription primers on gel beads, and dNTP substrates interacted. This process generated cDNA molecules with barcode and Unique Molecular Identifier (UMI) information for subsequent sequencing. The SMART amplification method was employed for second strand synthesis. GEMs were broken to fragment cDNA molecules, which were then purified using magnetic beads. The purified cDNA was utilized as the template for Polymerase Chain Reaction (PCR) amplification. After amplification, the cDNA was chemically fragmented into 200-300bp fragments and underwent end repair and A-tailing. The next step involved constructing scRNA-seq libraries, which were subsequently subjected to quality control. Upon passing quality control, the cDNA libraries were directly sequenced on Illumina sequencers. Data analysis was then performed on the scRNA-seq datasets, including aligning sequencing reads to a reference genome, quantifying gene expression levels, identifying differentially expressed genes, and clustering analysis to identify distinct cell populations. Following library preparation, We performed sequencing of the scRNA-seq libraries on a NovaSeq6000 (Illumina) using paired-end 150bp sequencing. This high-throughput sequencing platform generated massive amounts of data, capturing the transcriptomic profiles of individual cells.

### 2.3 Preparation and sequencing of immunomodulatory library

The microfluidic chips enclosed gel beads, cells, and reverse transcriptase systems within oil droplets, which contained barcodes and UMIs. Each droplet’s gel beads disintegrated after encapsulation, causing the cells to cleave and release mRNA. The reverse transcriptase system facilitated the reverse transcription of the mRNA, resulting in the generation of cDNA with barcodes and UMIs. The cDNA was then divided into two portions. One part was used for the construction of the immuno-genomic library, which involved designing nested PCR primers in the C region of T-cell receptor (TCR) or BCR and enriching the TCR or BCR sequences. The other part was directly used for the construction of the 5’ transcriptome library. Leveraging the 10X Genomics platform, this methodology enables the simultaneous acquisition of data on 5’ gene expression in 500-10,000 single cells and the full-length sequences of the TCR/BCR, thereby enabling high-throughput sequencing of both gene transcripts and the immunomodulatory repertoire at the single-cell level.

### 2.4 Bioinformatics analysis of scRNA-seq

#### 2.4.1 Quality control and standardization of data

Initially, Illumina raw base call files (BCLs) obtained from Illumina sequencing were demultiplexed into FASTQ files using the 10X data “mkfastq” module of the Cell Ranger (version 5.0.0) pipeline. We employed the default and recommended parameters of the Cell Ranger “count” module to align and quantify the FASTQ sequences using the GRCh38 1.2.0 human reference genome. To create a consolidated feature-barcode matrix, multiple runs of the Cell Ranger “count” module were aggregated together. This matrix contained barcode, gene, and count values, with each entry representing the number of reads originating from a specific barcode and mapping to a particular gene. Aggregating the outputs from different runs ensured the combination of data from various samples into a single matrix, facilitating downstream analyses. The obtained feature-barcode matrix then underwent quality control measures using Seurat R package (version 4.1.3). The purpose of quality control was to remove low-quality cells that could introduce noise and confound the interpretation of results. Three filtering criteria were applied: The cells that exhibited over 0.1 expression of the mitochondrial genes were excluded, the cells that exhibited less than 200 or more than 6000 expressed genes were excluded, and genes found in fewer than three cells were eliminated. Following the application of these filtering criteria, 140,785 cells in total were saved for further examination. Before clustering, batch correction was performed using the Seurat R program to address any possible batch effects between samples. Correcting for batch effects ensured that differences in gene expression primarily resulted from biological factors rather than technical artifacts.

#### 2.4.2 Selection of highly variable genes, dimensionality reduction clustering and visualization of data

We normalized the filtered gene-barcode matrices using the “LongNormalize” technique. Normalization was essential to remove technical biases and ensure comparability of gene expression values across cells. In order to combine data from various samples, we utilized the concept of ‘anchors’. The Seurat package’s functions “FindIntegrationAnchors” and “IntegrateData” were utilized to identify pairs of anchor points between the reference data set and the query data set. The Seurat package’s “FindVariableFeatures” function calculated the mean and variance for each gene and considered their relationship. We kept only the genes that influenced group variability, while managing the connection between average expression and variability. The most important sources of variation were retained while the dimensionality of the high-dimensional gene expression data was reduced using principal component analysis (PCA). An elbow plot was employed to determine the optimal number of components to retain, and the data was reduced to its top 20 principal components. We utilized the “FindClusters” function from the Seurat package to perform clustering with a resolution of 0.5. Cells were grouped according to their similarity in gene expression profiles, allowing to identify different cell populations. The uniform manifold approximation and projection (UMAP) algorithm was employed to visualize the clusters on a 2D map. Based on the clustering results, 20 final cell clusters were determined. To explore cellular heterogeneity within specific cell types, sub-clustering was performed. We performed the identical procedure on specific data subsets, typically confined to a single cell type.

#### 2.4.3 Cell type identification, differential gene screening, and functional enrichment analysis

We utilized the “FindAllMarkers” function to identify marker genes and differentially expressed genes (DEGs) for each cluster. DEGs were genes that showed significant expression differences between cells within the target cluster and cells in all other clusters. The expression profiles of cells from the target cluster were compared with those from other clusters using the Wilcoxon rank sum test. Differential expression analysis was performed by treating cells within each cluster as duplicates and subjecting them to statistical tests. The “FindAllMarkers” function implemented three parameters to determine marker genes: a) a log-fold change threshold greater than 0.25, b) a minimum difference percentage greater than 0.25, and c) a minimum expression percentage greater than 0.1. The *p*-value of each gene was calculated to assess its significance within each cell cluster. By utilizing this information, it became possible to infer the cell types of different clusters and visualize the cells with their corresponding cell type labels. We utilized the ClusterRprofile R package (v4.2.2) for Kyoto Encyclopedia of Genes and Genomes (KEGG) and Gene Ontology (GO) analysis.

#### 2.4.4 Pseudotime analysis

For pseudotime analysis, we employed the “DDRTree” algorithm from Monocle 2, which utilizes reverse graph embedding. In this approach, each cell was considered a point in a high-dimensional space, with each dimension corresponding to the expression level of an ordered gene. By projecting the data onto a lower-dimensional space, we employed PCA to reduce the dimensionality of the data. Subsequently, Monocle 2 constructed a spanning tree, where cells were moved to the nearest vertices in the tree and the positions of the vertices were continuously updated to accommodate cells. Through iterative processes, the spanning tree and cell positions eventually converged. Once the spanning tree was established and a vertex was selected as the root, Monocle 2 calculated the pseudotime of each cell by measuring its geodesic distance along the tree from the root. This allowed for the determination of the order in which cells progressed in a biological process. Furthermore, based on the main graph, cells could be automatically assigned to different branches, reflecting diverse developmental trajectories or functional states.

#### 2.4.5 TF activity inference

We analyzed the assessment of Transcription Factors (TFs) activity using the DoRothEA gene set. The initial step in this evaluation involves using the run_Viper function to process the regulons of DoRothEA and determine the TF activity. This activity is computed based on the mRNA expression level of the TF’s target. The credibility of the interaction between TFs and their targets is classified into five levels (A-E), with A being the most credible and decreasing in credibility with each subsequent level. Subsequently, TF regulons labeled as ‘A’, ‘B’, and ‘C’ are selected for high confidence to enhance the reliability of the subsequent analysis. These selected TF regulons are then utilized to compute Viper scores, which are scaled and incorporated as the ‘Dorothea’ slot in the integrated Seurat object. The scaled Viper scores are calculated for each severity group. Moreover, TFs are ranked based on the variance of their corresponding Viper scores to identify TFs with the most significant variability in activity. To aid visualization, the top 60 TFs with the highest variability in scores per severity group are selected, resulting in the visualization and in-depth exploration of a total of 180 TFs.

#### 2.4.6 Data analysis and visualization of BCR and TCR

The sequencing data underwent quality control and filtering to eliminate low-quality reads and contaminants. Steps such as primer trimming and duplicate sequence merging were applied to ensure data cleanliness. The preprocessed reads were aligned to a reference genome or sequence database to retrieve BCR or TCR sequences. From the alignment results, we extracted key features of BCR or TCR, such as V (Variable) and J (Joining) gene usage, CDR3 (Complementarity Determining Region 3) length, and amino acid sequences. Clustering was then performed on similar BCR or TCR sequences to identify clonal types, which represent sequences with identical V and J genes, CDR3 sequences, and other features. Further analysis focused on each clonal type, including frequency calculation, diversity assessment, and examination of relationships between different clonal types. Functional annotation was carried out to gain insights into the structural, affinity, and antigen specificity characteristics of the clonal types. The analysis results were visualized through various plots, such as clonal abundance distribution, diversity curves, and clonal network graphs. These visualizations provide a comprehensive understanding of the BCR or TCR repertoire and its functional properties.

### 2.5 Flow cytometric analysis

The laboratory promptly processed the peripheral blood samples upon their arrival. To reduce non-specific antibody binding, the blood cells were pre-incubated for 10 min with a human Fc receptor blocking reagent (miltenyibiotec, 5220901568). The blood cells were then separated into three panels to detect distinct subsets of PBMCs. Panel 1 was used to identify monocytes and major lymphocyte populations, specifically CD3+CD4+ T cells, CD3+CD8+ T cells, CD19+ B cells, and CD3-CD16+/CD56+ NK cells, using the Agilent TBNK 6 color immunotyping kit (Cat# 8920251). In Panel 2, we concentrated on B-cell subsets, identifying plasma cells using BD CD19-PE (Cat# 340720), BD CD24-BV510 (Cat# 563035), and BD CD38-APC (Cat# 340677). Panel 3 aimed to analyze monocyte subsets, specifically CD14+CD16- classical monocytes, where EDTA-anticoagulated whole blood was incubated with a combination of monoclonal antibodies: BD CD45-PerCP (Cat# 340665), BD CD14-PE (Cat# 340683), BD CD16-APC (Cat# 561304), and BD CD24- BV510 (Cat# 563035). After a 15-minute staining incubation in the dark at room temperature, we lysed the red blood cells with 1 ml of RBC lysis buffer for 10 minutes. The cells were then put onto the ACEA Novocyte D2040R after being cleaned three times with one milliliter of PBS. Approximately 1 × 10^4 cells were collected by the flow cytometer. We used the ACEA Novocyte software to gate the cell populations.

### 2.6 Measurement of cytokines and chemokines

We used the testing reagents provided by Qingdao Raisecare Biotechnology Co., Ltd. (Shandong, China) to identify IFN-α, IFN-γ, IL-1β, IL-2, IL-4, IL-5, IL-6, IL-8, IL-10, IL-12P70, IL-17, and TNF-α, through multi-microsphere flow immunofluorescence assay. We used an automatic flow cytometer (Raisecare) to analyze the obtained data.

### 2.7 Statistical analysis

R version 4.1.3 was used for the statistical analysis. In this analysis, we used the Levene’s test and Shapiro-Wilk normality test to assess the normality of data and the equality of variances before applying certain tests. If the data failed to meet the assumption of normality (*p*< 0.05), it was recommended to use the Wilcoxon rank sum test, a non-parametric method. Conversely, if the data followed a normal distribution, the t-test could be used. For paired samples, the paired U test or paired t-test should be employed. Spearman correlation analysis was utilized to examine the association between immune cells and the Expanded Disability Status Scale (EDSS) scores, whereas the Analysis of Variance (ANOVA) was applied to determine whether the percentage differences of immune cells under different attack manifestations were statistically significant.

## 3 Results

### 3.1 Study design and major changes in single-cell RNA profiling of PBMCs between NMOSD patients and healthy controls

Twelve recently drawn peripheral blood samples from six individuals with NMOSD (N1-N6) were acquired based on the criteria established by the International Panel for NMO Diagnosis (15). Blood was collected twice from each patient - once prior to and once ten days after steroid therapy. As controls, we obtained peripheral blood samples from five donors in good health (H1-H5) of similar age (**Supplementary Table 1**).

We conducted scRNA-seq analysis on PBMCs that were isolated from our samples using the 10X genomics platform. After ensuring quality control, we analyzed a total of 140,785 cells, consisting of 33,044 cells from NMOSD patients before steroid therapy (NBs), 54,616 cells after steroid therapy (NAs), and 53,125 cells from HCs (**Supplementary Table 2**). Additionally, we retained information about BCR and TCR. We detected 27,848 cells with BCR signatures (IGH) and 69,792 cells with TCR signatures (TCRα-β pair) (**Supplementary Tables 3**, **4**). Of these, we identified 18,908 B cells that had a single productive IGH allele (4,267 from NBs, 10,104 from NAs, and 4,537 from HCs) and 53,204 T cells that had a single productive TCRα-β pair (10,603 from NBs, 15,405 from NAs, and 27,196 from HCs).

**Figure 1:**
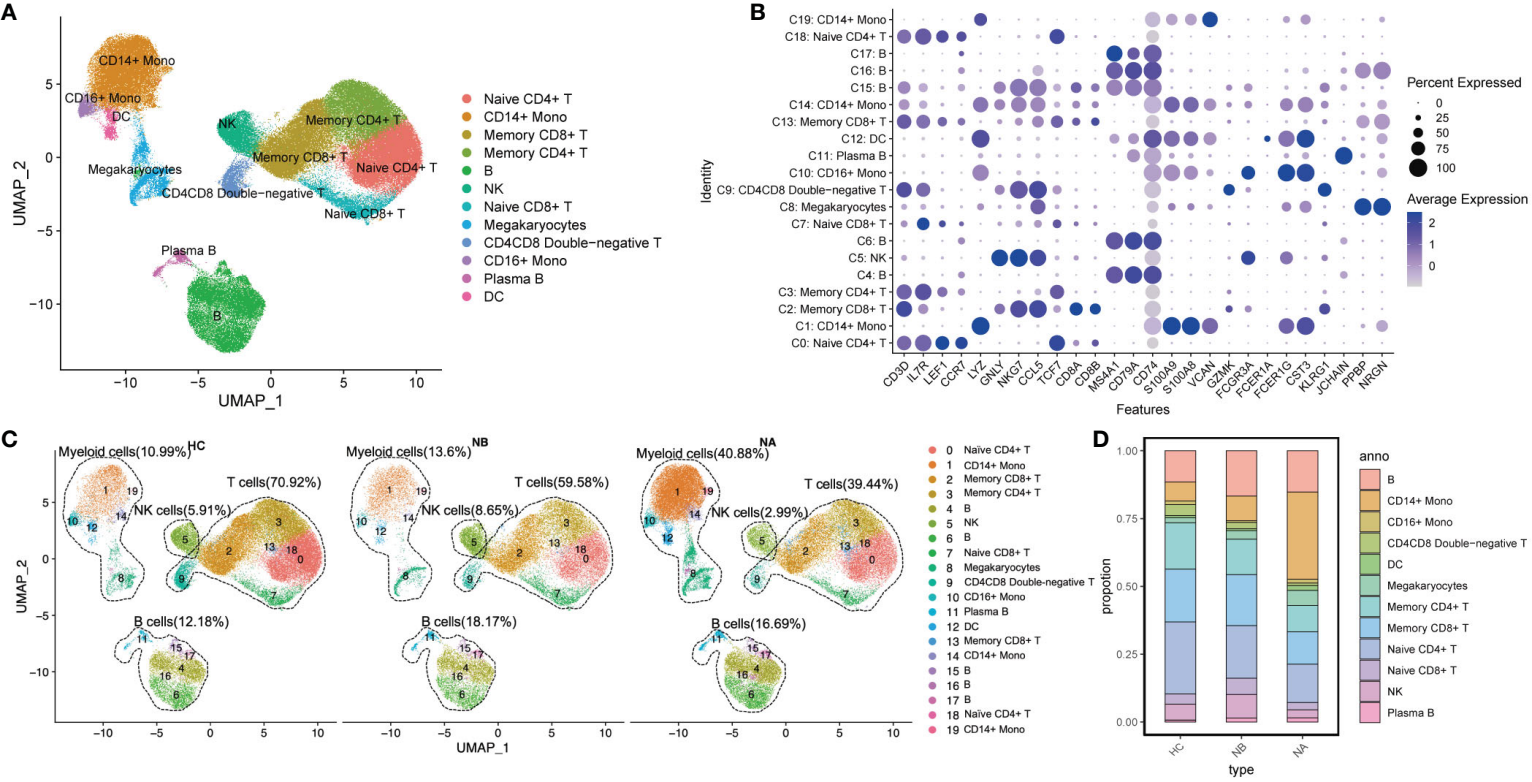
Single-cell analysis of PBMCs from NMOSD patients and HCs: **(A)** UMAP presentation of major cell types in PBMCs; **(B)** A dot plot showing expression of canonical maker genes by different cell clusters; **(C)** UMAP projection showing the distribution of 12 major cell groups among HCs (n=5) and NMOSD patients (NB, n=6; NA, n=6); **(D)** Proportion of major cell types shown in **(A)** separated by condition.

We divided the PBMCs into 20 clusters (**Supplementary Figure 1**) and identified the key cell subtypes that were present in the samples. These included CD4+ T cells, CD8+ T cells, CD4-CD8- T cells, B cells, natural killer (NK) cells, CD14+ monocytes, CD16+ monocytes, dendritic cells (DCs), and megakaryocytes (Mgk) (**Figures 1A, B**). We categorized our dataset into 12 distinct cell types (**Figure 1C**), which included naïve CD4+ T cells (CD4, LEF1, CCR7, 20.08%), memory CD4+ T cells (CD4, IL7R, TCF7, 13.26%), naïve CD8+ T cells (CD8B, LEF1, CCR7, 3.86%), memory CD8+ T cells (CD8A, GZMK, CCL5 or CD8B, TCF7, 16.37%), CD4CD8 double-negative T cells (KLRG1, CD3D, CD4-, CD8-, 2.48%), B cells (CD79A, MS4A1, CD74, 14.15%), plasma B cells (JCHAIN, 1.18%), NK cells (GNLY, NKG7, 5.42%), CD14+ monocytes (S100A8, S100A9, LYZ or VCAN, 17.25%), CD16+ monocytes (FCER1G, FCER3A, 1.21%), DCs (CST3, CD74, FCER1A, 1.10%), and Mgk (PPBP, NRGN, 3.64%).

We performed functional enrichment analysis of the significantly DEGs between each cluster. GO analysis revealed that clusters 4, 6, 15, 16, and 17 were associated with immunoglobulin-mediated immune response and B cell-mediated immunity (**Supplementary Figure 2**). Clusters 0, 2, 3, 7, 9, 13, and 18 were linked to T-cell activation and differentiation. Clusters 1, 5, 10, 12, 14, and 19 were associated with the regulation of innate immune response, myeloid leukocyte activation, and differentiation. Additionally, cluster 11 was associated with humoral immune response mediated by circulating immunoglobulin, while cluster 8 was associated with platelet activation and hemostasis.

We conducted a comparison of pooled PBMCs from NBs to HCs, and as observed (**Figures 1C, D**), the percentage of B cells, myeloid cells, and NK cells increased, while the proportion of T cells decreased. Additionally, when we compared the subtype representations between NBs and NAs, we observed that the percentage of myeloid cells increased in NAs, whereas B cells, T cells, and NK cells decreased. The percentage of NK cells in NAs was even lower than in HCs.

### 3.2 Changes of major immune cell types

**Figure 2:**
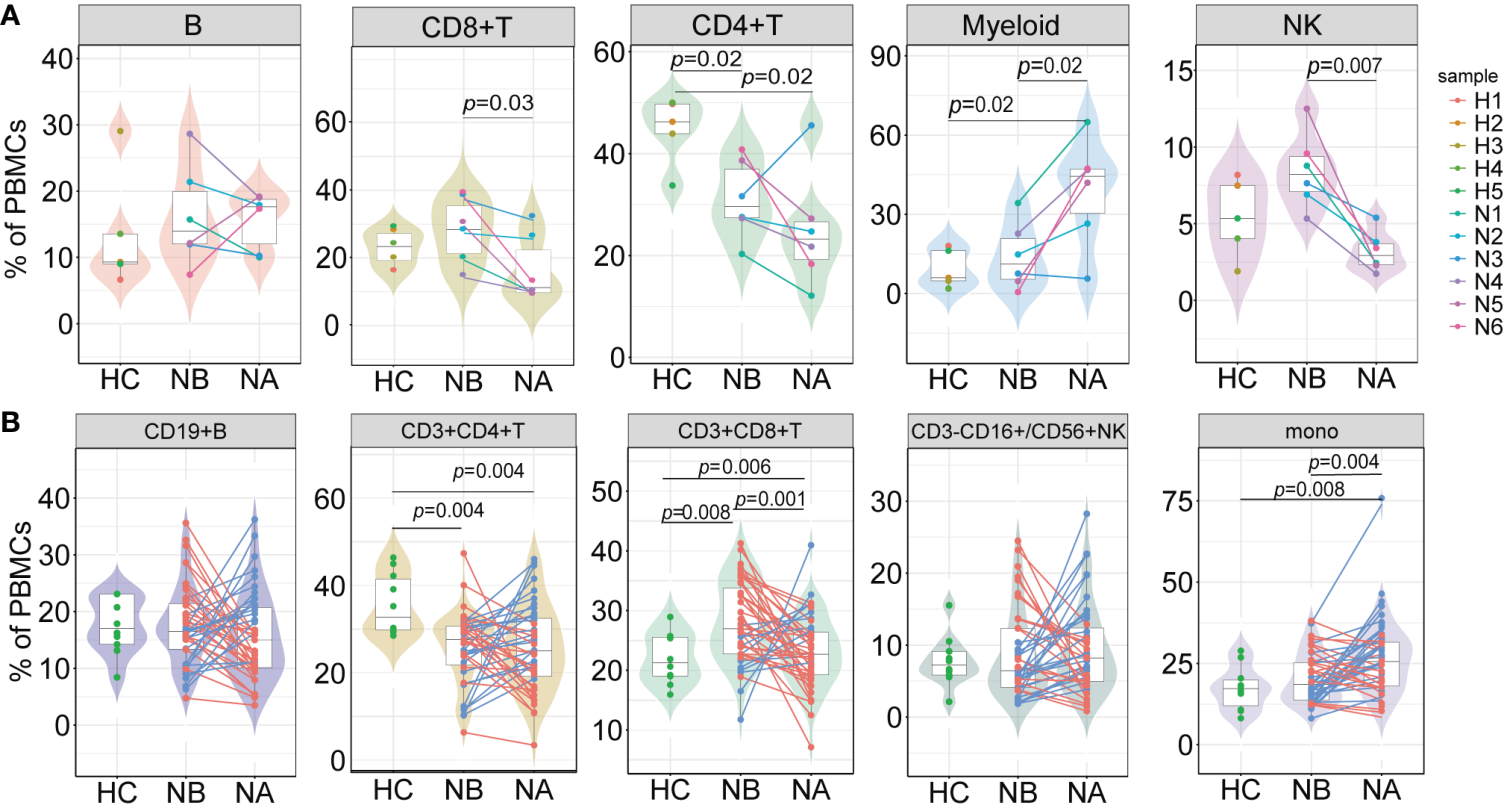
Differences in the proportions of major cell types across conditions. **(A)** The proportion differences of each cell type in PBMCs under different conditions revealed by scRNA-seq (NB, n = 6; NA, n = 6; HCs, n = 5). The samples are color-coded based on donors. *P*-values are calculated using paired t-tests to compare NBs and NAs. *P*-values between NBs or NAs and HCs are calculated using Mann-Whitney U-test; **(B)** The proportion differences of each cell type under different conditions revealed by flow cytometric validation (NB, n = 43; NA, n = 43; HCs, n = 10). The patients’ post-therapy changes are color-coded. *P*-values between NBs and NAs are calculated using the paired U-test. *P*-values between NBs or NAs and HCs are calculated using Mann-Whitney U-tests.

At the individual level, we conducted pairwise comparisons using scRNA-seq data to analyze the variations in immune cell types among different groups (**Figure 2A**). We also conducted flow cytometry analysis on 43 NMOSD patients and 10 HCs (**Figure 2B**; **Supplementary Figure 3A**). Analysis of scRNA-seq data revealed that NBs had a notably higher proportion of CD8+ T cells (*p* = 0.03, Wilcoxon signed rank test) and NK cells (*p* = 0.007, paired t-test) and a lower proportion of myeloid cells (*p* = 0.02, paired t-test) in comparison to NAs (**Figure 2A**), which was consistent with the flow cytometric dates (**Figure 2B**). Moreover, NBs showed a significant increase in the percentage of CD8+ T cells compared to HCs (*p* = 0.008, Mann-Whitney U-test), which rapidly declined after therapy (*p* = 0.001, paired U-test). ScRNA-seq data indicated a lower percentage of CD4+ T cells in NBs compared to HCs (*p* = 0.02, Mann-Whitney U-test, **Figure 2A**), which aligned with our flow cytometric findings (*p* = 0.004, Mann-Whitney U-test, **Figure 2B**). Furthermore, scRNA-seq data demonstrated a higher percentage of myeloid cells (*p* = 0.02, Mann-Whitney U-test) and a lower percentage of CD4+ T cells (*p* = 0.02, Mann-Whitney U-test) in NAs than in HCs (**Figure 2A**). Flow cytometric data (**Figure 2B**) showed a similar pattern with monocyte cells (*p* = 0.008, Mann-Whitney U-test) and CD4+ T cells (*p* = 0.004, Mann-Whitney U-test). Spearman’s correlation analysis showed that CD4+ T cells (*R* = 0.065, *p* = 0.680), monocytes (*R* = −0.071, *p* = 0.653) in NBs, and B cells (*R* = 0.058, *p* = 0.710), CD4+ T cells (*R* = 0.098, *p* = 0.532) in NAs moderately correlated with the EDSS scores (**Supplementary Figures 4A, B**). There were no appreciable variations in the kinds of immune cells between the various attack manifestations in NMOSD patients (**Supplementary Figure 5**).

### 3.3 B cell clustering and BCR repertoires

**Figure 3:**
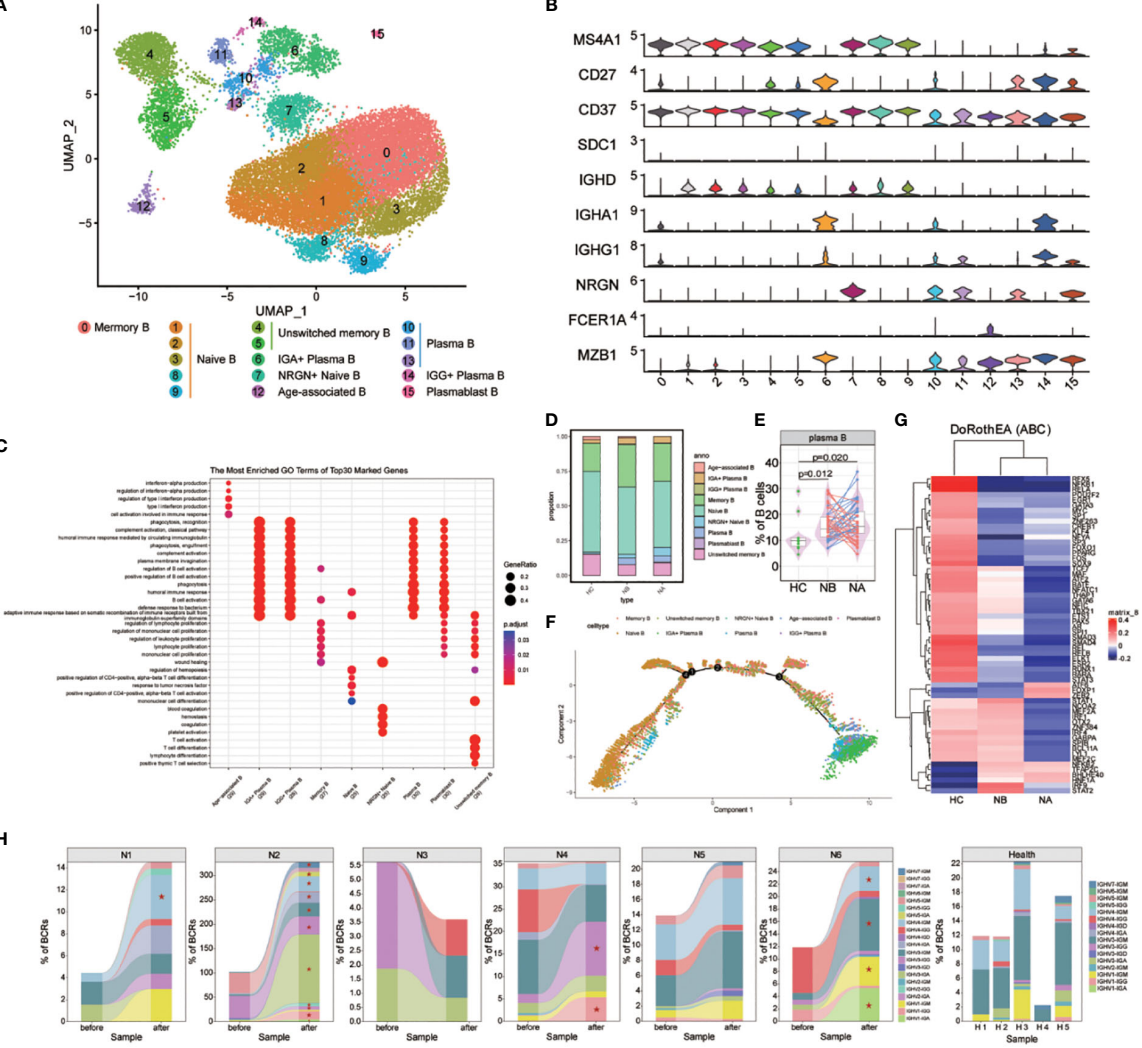
Heterogeneity of high-resolution features of B cells between NMOSD patients and HCs. **(A)** UMAP visualization of heterogeneous B cell clusters based on genetic markers; **(B)** Violin plots showing expression of canonical maker genes for B cell clusters; **(C)** GO analysis of B cell clusters; **(D)** Proportion of B cell types shown in **(A)** separated by condition; **(E)** The proportion of plasma cells revealed by flow cytometric validation (NB, n = 43; NA, n = 43; HCs, n = 10); **(F)** Trajectory analysis of B cells; **(G)** Heatmap of top 60 highly variable TF activities among NMOSD patients and HCs; color-coded by z-scores; **(H)** Tracking of BCR clonotype. Clonotype composition displayed using stacked bar plots, with different colors indicating IGHV families. Comparison between NBs and NAs is conducted using the two-sided Fisher’s exact test, with individual clonotypes marked with a star if FDR<0.01. Only clonotypes with a size ≥3 are plotted.

We analyzed the subtypes of B cells in greater detail using bioinformatics, which led to the identification of 16 distinct sub-clusters (**Figure 3A**). We used genetic markers to identify different B cell subtypes: naive B cells in clusters 1, 2, 3, 8, and 9, NRGN+ naive B cells in cluster 7, memory B cells in cluster 0, unswitched memory B cells in clusters 4 and 5, plasmablast B cells in cluster 15, plasma B cells in clusters 10, 11, and 13, IGG+ plasma B cells in cluster 14, IGA+ plasma B cells in cluster 6, and age-associated B cells in cluster 12 (**Figure 3B**). We then assessed the DEGs in B cell clusters (**Figure 3C**). GO analysis indicated that naive B cells were associated with differentiation of mononuclear cells and T cells, as well as the response to tumor necrosis factor. Memory B cells were associated with the regulation of B cell activation, lymphocyte proliferation, and mononuclear cell proliferation. Meanwhile, plasma and plasmablast B cells were associated with humoral immune response mediated by circulating immunoglobulin, regulation of B cell and complement activation. Age-associated B cells were associated with the cell activation involved in the immune response, production of type I interferon (IFN), and interferon-alpha. Lastly, unswitched memory B cells were associated with the differentiation of mononuclear cells, T cells, and lymphocytes.

Differences were observed in the B cell compartments among NBs, NAs and HCs (**Figure 3D**). The small sample size for scRNA-seq precluded reaching statistical significance. Nonetheless, NBs had a higher proportion of IGG+ plasma B cells, IGA+ plasma B cells, plasma B cells, and memory B cells compared to HCs (**Supplementary Figure 6**). After steroids therapy, there was a decrease in the proportion of age-associated B cells and IGG+ plasma B cells, and an increase in plasmablast B cells and NRGN+ naive B cells. The changes in the proportion of plasma and memory B cells indicate that NMOSD induces a robust humoral immune response. To confirm these findings, flow cytometric analysis was conducted on an additional 43 NMOSD patients and 10 healthy controls (**Figure 3E**; **Supplementary Figure 3B**). The analysis showed that both NBs and NAs had higher proportion of plasma cells compared to HCs, as indicated by the *p*-values of 0.012 (Mann-Whitney U-test) for NBs and 0.02 (Mann-Whitney U-test) for NAs.

GO analysis revealed that, compared with HCs, DEGs enriched in NBs were primarily involved in the positive regulation of immunoglobulin production, including IGKV1-39, IGKV3-20 and IGLV4-69 (**Supplementary Figure 7**). Furthermore, the most highly overexpressed DEGs in NBs were associated with the response to type I interferon, including IFITM1, IRF7, ISG15, MX1, and OAS1. Following therapy, upregulated DEGs in the regulation of intrinsic apoptotic signaling pathway were observed, including XBP1, GPX1, HERPUD1, S100A8, and S100A9. Additionally, NAs exhibited overexpression of DEGs involved in the production of molecular mediators of immune response, such as IGLV1-44, IGLV2-23, IGLV2-14, IGKV1-12, and MZB1.

We also conducted trajectory analysis of B cells (**Figure 3F**; **Supplementary Figures 8**, **9**). In individuals with NMOSD, the differentiation pathways are similar to those in HCs, but with a higher proportion of cells differentiating into memory, IGA+ plasma B, IGG+ plasma B, and plasma B cells. The analysis also revealed a stepwise reduction in the expression of CD37, IGHD, MS4A1 and NRGN, along with an upregulation of IGHA1, IGHG1, MZB1 and SDC1 (**Supplementary Figure 10**). Notably, the proliferative maker MZB1 and the emigrant marker SDC1 were highly expressed in IGA+ plasma B, IGG+ plasma B and plasma B cells. In the process of seeking upstream regulatory mechanisms, we used the Dorothea algorithm and the Viper inference tool to infer TF activity and score the activity of each regulon (16, 17). In NBs, we found increased activity scores for STAT1, STAT2, IRF1, IRF4, IRF9, and NFKB2, which are known to be involved in the transcription cycle of interferon-stimulated genes (ISGs), indicative of heightened activation of NFKB/STAT signaling (**Figure 3G**). Additionally, we observed a decrease in IRF9 and STAT2 TF activities following steroid therapy (**Figure 3G**).

The scBCR-seq data were analyzed to investigate BCR repertoires dynamics. Compared to HCs, NMOSD patients show an increase in the proportions of IGHG and IGHA (**Supplementary Figures 11**, **12**). The assessment of BCR clonality in each patient (**Figure 3H**) revealed a slight increase in the percentage of clonal BCRs in NA compared to NB and HC, as shown in the supplementary material (**Supplementary Figure 13**). Oligoclonal expansions, primarily consisting of IgA and IgG isotypes, were observed in all NAs except N3 (**Figure 3H**). The usage of IGHV genes in the expanded clones varied among NMOSD patients (**Figure 3H**), and no significant bias was found between NMOSD patients and HCs (**Supplementary Figure 14**). Moreover, there was a substantial increase in the clonal BCR quantity in memory B cells, naive B cells, and plasma B cells (**Supplementary Figure 15**). Overall, our data suggests that NMOSD patients show extensive expansion and transcriptional homogeneity, triggering B-cell reactions with specific antibodies during the acute phase instead of responding to superantigens.

### 3.4 T cell subsets analysis and TCR repertoires

**Figure 4:**
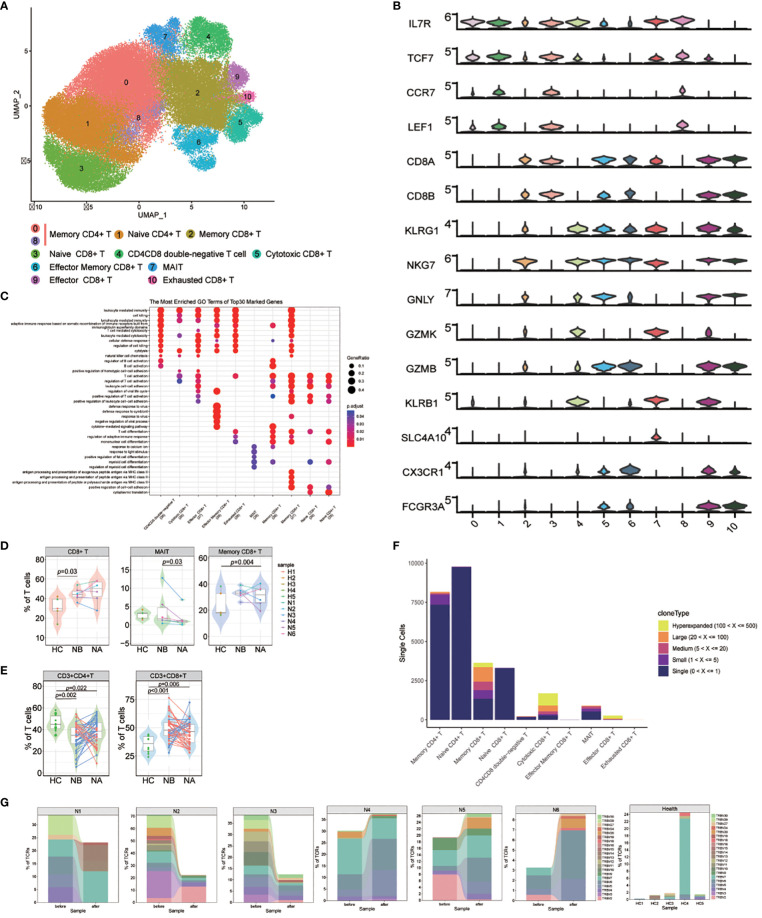
Heterogeneity of high-resolution features of T cells between NMOSD patients and HCs. **(A)** UMAP visualization of heterogeneous T cell clusters based on genetic markers; **(B)** Violin plots showing expression of canonical maker genes for T cell clusters; **(C)** GO analysis of T cell clusters; **(D)** Percentage of CD8+ T, MAIT and memory CD8+ T cells revealed by scRNA-seq (NB, n = 6; NA, n = 6; HCs, n = 5). The samples are color-coded based on donors. *P*-values are calculated using the Wilcoxon signed rank test to compare NBs and NAs. *P*-values between NBs or NAs and HCs are calculated using the Mann-Whitney U-test; **(E)** Percentage of CD4+ and CD8+ T cells determined through flow cytometric validation (NB, n = 43; NA, n = 43; HCs, n = 10); **(F)** TCR clonotype tracking in T cell subsets.; **(G)** Tracking of TCR clonotype. Clonotype composition displayed using stacked bar plots, with different colors indicating TRBV families. Comparison between NBs and NAs is conducted using the two-sided Fisher’s exact test, with individual clonotypes marked with a star if FDR<0.01. Only clonotypes with a size ≥3 are plotted.

We named 10 T cell subsets by identifying marker gene expressions (**Figures 4A, B**). These subsets included naive CD4+ T cells (cluster 1), naive CD8+ T cells (cluster 3), memory CD4+ T cells (cluster 0 and 8), memory CD8+ T cells (cluster 2), effector memory CD8+ T cells (cluster 6), cytotoxic CD8+ T cells (cluster 5), exhausted CD8+ T cells (cluster 10), effector CD8+ T cells (cluster 9), CD4CD8 double-negative T cells (cluster 4), and mucosal-associated invariant T (MAIT) cells (cluster 7). GO analysis (**Figure 4C**) revealed that naive CD4+ and CD8+ T cells were associated with T cell activation and differentiation, as well as positive regulation of cell-cell adhesion. Memory CD4+ T cells were associated with the cytokine-mediated signaling pathway and the regulation of adaptive immune response. CD8+ T cells were associated with cell killing, cytolysis, and leukocyte-mediated cytotoxicity. Notably, memory CD8+ T cells were also associated with antigen processing and presentation of peptide antigens via MHC class II. Effector memory CD8+ T cells were primarily associated with response to viruses. Finally, MAIT cells were found to be correlated with positive regulation of fat cell differentiation and myeloid cell differentiation.

Our findings showed that NMOSD patients had a decreased proportion of T cells in PBMCs compared to HCs. However, compared to HCs, the proportion of CD8+ T cells in T cell population is notably increased (*p* = 0.03, Mann-Whitney U-test, **Figure 4D**; **Supplementary Figure 16**). Additionally, the proportion of MAIT cells was substantially increased in NAs compared to NBs (*p* = 0.03, Wilcoxon signed rank test), and memory CD8+ T cells were increased in NAs compared to HCs (*p* = 0.004, Mann-Whitney U-test). These results suggest that the reduction of T cells in PBMCs may primarily be due to a decrease in CD4+ T cells, which play a pivotal role in the activation and regulation of other immune cells. Furthermore, flow cytometric analysis of T cell subsets in 43 NMOSD patients confirmed higher proportion of CD8+ T cells (*p* < 0.001, Mann-Whitney U-test) and lower proportion of CD4+ T cells (*p* = 0.002, Mann-Whitney U-test) compared to HCs, with no significant changes observed following therapy (**Figure 4E**).

Because of the critical role of T cells in adaptive immunity, the transcriptomic changes were analyzed between the NMOSD patients and the HCs. GO analysis demonstrated that many upregulated DEGs positively correlated with the response to type I interferon in NBs compared to NAs and HCs, including IRF7, ISG15, MX1, and SP100 (**Supplementary Figure 17**). Additionally, DEGs related to the response to viral infections, such as DDIT4, CXCR4, EIF5A, TNFAIP3, BCL2, and IRF1, were also upregulated in NBs. Following therapy, we observed an increase in the expression of genes involved in immune response-activating signaling pathways and lymphocyte differentiation. KEGG analysis further revealed significant changes in key gene sets, including antigen processing and presentation, T cell receptor signaling pathway, and NF-kappa B signaling pathway in NAs (**Supplementary Figure 18**).

For a deeper grasp of the clonal relationships among T cell subsets in NMOSD, we analyzed the TCR of 10 subsets. Significant clonal expansion was detected in memory CD8+ T cells, cytotoxic CD8+ T cells, effector CD8+ T cells, and MAIT cells (**Figure 4F**). Assessment of TCR clonality in each patient (**Supplementary Figure 19**) revealed a lower percentage of clonal TCRs in NAs compared to NBs (*p*=0.04, Wilcoxon rank-sum test). We then investigated whether shared expanded clones exist among NBs, NAs, and HCs, and found that most of these expanded clones exhibited individual variability (**Figure 4G**).

### 3.5 Myeloid subsets and responses

**Figure 5:**
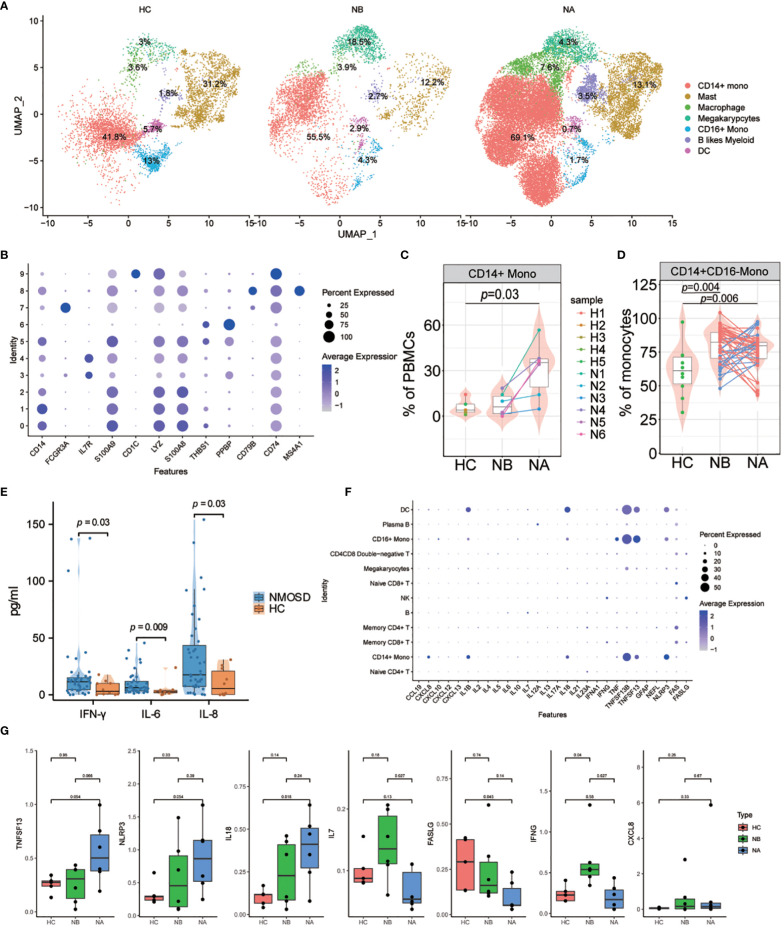
Heterogeneity of high-resolution features of myeloid cells between NMOSD patients and HCs. **(A)** UMAP projection showing the distribution of 7 myeloid cell groups among HCs (n=5) and NMOSD patients (NB, n=6; NA, n=6); **(B)** Violin plots showing expression of canonical maker genes for myeloid cell clusters; **(C)** Percentage of CD14+ monocytes revealed by scRNA-seq (NB, n = 6; NA, n = 6; HCs, n = 5). The samples are colored according to donors. *P*-values between NAs and HCs are calculated using the Mann-Whitney U-test; **(D)** Percentage of CD14 monocytes determined through flow cytometric validation (NB, n = 43; NA, n = 43; HCs, n = 10); **(E)** Comparison of the levels of IFN-γ, IL-6, and IL-8 in the peripheral blood of 43 NMOSD patients and 10 HCs. *P*-values are adjusted by false discovery rate; **(F)** Dot plots of gene expression of molecular biomarker genes for populations shown in NMOSD; **(G)** Comparison of the expression levels of CXCL8, IL7, IL18, TNFSF13, IFNG, NLRP3, and FASLG between HCs (n=5), NBs (n=6), and NAs (n=6).

To further our understanding of transcriptomic changes in myeloid cells among NMOSD patients, we performed a quantitative evaluation of these changes across all clusters. The myeloid cell compartment included monocytes, DCs, macrophages(MΦ) and granulocytes (18). Myeloid cells were sub-grouped into 10 clusters (**Supplementary Figure 20**). Seven distinct subsets of myeloid cells were identified: CD14+ monocytes (CD14, 62.35%), CD16+ monocytes (FCGR3A, 4.04%), MΦ (THBS1, 6.35%), DCs (CD1C, 1.90%), mast cells (IL7R, 16.19%), B-like myeloid cells (MS4A1, 3.11%), and Mgk (PPBP, 6.05%) (**Figures 5A, B**). Following steroid therapy, we observed a significant increase (*p* = 0.03, Wilcoxon signed-rank test) in the percentage of CD14+ monocytes among the patients (**Figure 5C**; **Supplementary Figure 21**). Moreover, the proportion of CD14+ monocytes was significantly higher in NAs than in HCs (*p* = 0.03, Mann–Whitney U-test), with no significant difference observed between NBs and HCs. Furthermore, flow cytometric analysis confirmed that both NBs (*p* = 0.004, Mann-Whitney U-test) and NAs (*p* = 0.006, Mann–Whitney U-test) had a higher percentage of CD14+CD16- monocytes compared to HCs (**Figure 5D**; **Supplementary Figure 3C**).

We carried out an analysis of the transcriptomic changes in myeloid cells of NMOSD patients compared to HCs, with a focus on functional annotation using GO and KEGG analysis. Our analysis revealed important disparities between the two groups regarding astrocyte development and differentiation, granulocyte chemotaxis and migration, and chronic inflammatory response (**Supplementary Figure 22**). Specifically, we identified several upregulated DEGs associated with astrocyte development and differentiation in NAs when compared to NBs and HCs, including MT1X, VIM, S100A8, and S100A9. Furthermore, many DEGs related to granulocyte chemotaxis and migration and chronic inflammatory response were found to be upregulated in NAs, including CXCL8, S100A12, and THBS1. In addition, our KEGG analysis revealed that NMOSD patients exhibited an enrichment of motor proteins, focal adhesion, interactions of ECM receptors and the IL-17 signaling pathway (**Supplementary Figure 23**).

Finally, we assessed the cytokine and chemokine levels in the peripheral blood of NMOSD patients (**Supplementary Table 1**). Compared with HCs, NMOSD patients had exhibited elevated levels of the inflammatory cytokines IFN-γ, IL-6, IL-8 (**Figure 5E**; **Supplementary Figure 24**). Moreover, we examined the expression levels of inflammatory cytokines and chemokines, along with molecular biomarkers, in the patients (**Figure 5F**). Our analysis revealed that B cells predominantly express IL6 and IL7, whereas myeloid cells predominantly express CXCL8, IL1B, IL18, TNF, TNFSF13B, TNFSF13, and NLRP3; T and NK cells, on the other hand, mainly express IFNG, FAS, and FASLG (**Figure 5F**). Comparing the expression levels of these molecules in NMOSD patients versus HCs, we found that there was an increase in CXCL8, IL7, IL18, TNFSF13, IFNG and NLRP3 expression, but a decrease in FASLG expression (**Figure 5G**). Moreover, we found that CXCL8 expression was particularly strong in CD14+ monocytes, while TNF expression was stronger in CD16+ monocytes. Expression of IL1B and IL18 was highest in DCs, while TNFSF13B, TNFSF13 and NLRP3 show higher expression in monocytes, DCs, and macrophages (**Supplementary Figure 25**). The above findings indicate that the greater abundance of myeloid cells is associated with heightened production of cytokines and chemokines in NMOSDs.

## 4 Discussion

Autoimmune inflammation is widely recognized as a key factor in the onset of NMOSD (19). Despite significant research and treatment efforts, there are still crucial questions that need to be resolved, such as identifying the causes of the disease and understanding the formation of immune checkpoint defects. While studies have focused on the pathogenic mechanisms mediated by B cells and T cells, the roles of the innate immune response have been overlooked (20–22). Hence, further investigation of the peripheral blood immune system in NMOSD is essential for identifying new potential targets for intervention.

In this study, we employed scRNA-seq to comprehensively investigate the cellular transcriptional changes in PBMCs of patients with NMOSD. This study presents the initial detailed transcriptional map of PBMCs and systematically investigates the immune cell diversity and tolerance impairment in patients with NMOSD. Our findings revealed major cell clusters and disease-specific subsets, enabling the identification of several signatures and crucial inflammatory pathways associated with NMOSD.

We performed scRNA-seq on a total of 140,785 cells, followed by cell annotation, DEG and pathway analysis. At this level of resolution, we identified 20 distinct cell clusters. Substantial cell abundance changes were noted during the acute phase of NMOSD onset and post-steroid treatment, with confirmation via flow cytometry and alignment with prior studies (23–25). These findings indicate crucial alterations in immune cells and widespread changes in cell proportions in NMOSD patients. Apart from the differentiation and activation of lymphocytes, we have also identified an expansion of myeloid cells and an excessive inflammatory response in innate immunity.

The significant success of B cell depletion therapy in treating NMOSD has confirmed the crucial role of B cells in disease progression (26, 27). Regarding to B cell populations, we observed similar total abundance of B cells in NMOSD patients compared to HCs. However, we observed a significant increase in the proportion of plasma cells and memory B cells among the B cell population in NMOSD patients. Additionally, we observed oligoclonal expansions mainly consisting of IgA and IgG isotypes of BCRs, further supporting the crucial role of B cells in the pathogenesis of NMOSD. The highly enriched immunoglobulin production pathway in NMOSD patients also suggested the expansion of plasma cells. These findings suggest that NMOSD may result from acquired immune response to specific conventional antigens instead of superantigens (7). Furthermore, the distinct patterns of response to type I interferon between NBs and NAs suggest complex changes in the interferon response at different stages of NMOSD (28). Specifically, it has been found that type I interferon drives memory B cells in NMOSD to generate large quantities of IL-6 (29). Following therapy, we observed upregulated DEGs including S100A8 and S100A9, which are crucial for regulating inflammatory response by stimulating leukocyte recruitment and inducing cytokine secretion (30). Overall, our findings provide compelling evidence for the involvement of B cells, particularly plasma B cells and memory B cells, in the pathogenesis of NMOSD. Moreover, the distinct interferon response patterns and upregulated DEGs suggest potential targets for therapeutic interventions in NMOSD.

Contrary to our expectations, NMOSD patients had fewer T cells than HCs. However, within the T cell population, NMOSD patients demonstrated a larger proportion of CD8+ T cells, particularly in the effector/memory subset, which is consistent with previous research (22). While CD4+ T cells have been traditionally regarded as pivotal in NMOSD and multiple sclerosis (MS) pathology, recent studies in human patients have demonstrated that CD8+ T cells instead dominate in MS lesions and display an activated cytotoxic phenotype (31, 32). Autopsy findings confirmed the infiltration of CD3+ and CD8+ T lymphocytes in perivascular lesions of NMO patients (33). Moreover, DEGs associated with the response to viral infections were upregulated in NMOSD patients, supporting the hypothesis that viral infections may trigger NMOSD activity (1). We also observed that DEGs positively associated with the response to type I interferon were enriched in T cells in NBs. Excessive and persistent IFN signaling has been linked to multiple autoimmune diseases, including myositis, rheumatoid arthritis (RA), systemic lupus erythematosus (SLE), Sjogren’s syndrome, and systemic sclerosis (34). The highly overexpressed DEGs associated with the response to type I interferon in NMOSD patients present novel therapeutic opportunities for targeting IFN signaling in the treatment of NMOSD. KEGG analysis revealed significant changes in NF-kappa B signaling pathway in NAs. The activation and differentiation of T lymphocytes and innate immunity cells are contingent upon the NF-kappa B signaling pathway, which plays a significant role in regulating the expression of various pro-inflammatory genes (35). Thus, our findings highlight the critical role of NF-kappa B signaling in T cells for NMOSD. Additionally, we observed a notable rise in IFNG expression in circulating memory CD8+ T cells of NMOSD patients. IFNG encodes the pro-inflammatory cytokine IFN-γ. In summary, our findings indicate an association between the increased percentage of CD8+ T cells, known for their quick antigen response, and NMOSD occurrence.

We observed expansion and altered differentiation of CD14+ monocytes in myeloid cells. Various proinflammatory cytokines are secreted by CD14+ monocytes, which are essential for the early inflammatory response (36). In addition to astrocyte development and differentiation, granulocyte chemotaxis and migration, and chronic inflammatory response, patients with NMOSD also exhibited activated IL-17 signaling. It is well recognized that IL-17 signaling is essential for the development of autoimmune illnesses and that it is engaged in biological processes such as neutrophil infiltration into the central nervous system (CNS) and promotion of neuroinflammation (37, 38). Therefore, we believe that myeloid cells influence the development of NMOSD through promoting the secretion of inflammatory factors, neuroinflammatory responses, and other pathways.

Compared to HCs, NMOSD patients show elevated levels of the cytokines IFN-γ, IL-6, and IL-8 in peripheral blood, as well as increased expression levels of CXCL8, IL7, IL18, TNFSF13B, IFNG, and NLRP3. In the pathogenesis of NMOSD, IL-6 performs a variety of roles, such as boosting plasmablast survival, impairing the blood-brain barrier’s integrity and functionality, and boosting the activation and differentiation of proinflammatory T-lymphocytes (39). Furthermore, numerous studies have confirmed the association of certain cytokines/chemokines and related molecules, such as IL-6, IL-17, CXCL8, BAFF, and NLRP3, with clinical activity and long-term outcomes in NMOSD (40, 41). Collectively, cytokines and immunological markers have the potential to be utilized in NMOSD for predicting short-term outcomes and identifying patients at risk of experiencing new attacks.

Our study had several limitations. The first is that the comparatively small sample size restrict the scRNA-seq analysis. Additionally, our scRNA-seq dataset exclusively comprised female NMOSD patients, aimed at mitigating potential gender bias, but it might not capture the same pathological transcriptional changes observed in male patients. This study did not investigate the inflammatory responses in the cerebrospinal fluid of NMOSD patients. Furthermore, in flow cytometry analysis of monocyte subsets, we used panel 3 to categorize monocytes based on CD16 expression into classical and other monocytes. This allowed us to calculate the proportion of classical monocytes within the population. However, it’s worth noting that analyzing CD16+ monocytes was unfeasible through flow cytometry, potentially resulting in an incomplete characterization of the monocyte subsets in our study.

In conclusion, we have compiled a single-cell transcriptome map of NMOSD. Our findings highlight the differentiation and activation of lymphocytes, expansion of myeloid cells, and an overactive inflammatory response in innate immunity. Our study sheds light on the presence of disease-specific immune cell subsets in NMOSD, offering valuable insights into the underlying mechanisms of NMOSD and identifying potential new targets for intervention.

## Data availability statement

The datasets presented in this study can be found in online repositories. The names of the repository/repositories and accession number(s) can be found below: HRA005682 (https://bigd.big.ac.cn/gsa-human/browse/HRA005682).

## Ethics statement

The studies involving humans were approved by The medical ethics committee at the Henan Provincial People’s Hospital. The studies were conducted in accordance with the local legislation and institutional requirements. Written informed consent for participation in this study was provided by the participants’ legal guardians/next of kin.

## Author contributions

YJ: Conceptualization, Data curation, Formal analysis, Investigation, Methodology, Project administration, Supervision, Validation, Writing – original draft, Writing – review & editing. SD: Conceptualization, Data curation, Formal analysis, Methodology, Writing – original draft. RP: Data curation, Writing – original draft. LQ: Writing – review & editing. MZ: Writing – review & editing. HL: Writing – review & editing. XW: Data curation, Investigation, Writing – original draft. JZ: Project administration, Writing – review & editing. GP: Formal analysis, Methodology, Writing – original draft. YW: Data curation, Writing – original draft. WL: Funding acquisition, Project administration, Supervision, Writing – review & editing.
