# Supplementary material for: Single-cell RNA sequencing reveals cell type-specific immune regulation associated with human neuromyelitis optica spectrum disorder
Source: Front Immunol. 2024 Feb 19;15:1322125. doi: 10.3389/fimmu.2024.1322125 (PMC10909925; doi:10.3389/fimmu.2024.1322125)
Supplement: Supplementary file 1 [file DataSheet_1.docx]

***Supplementary Material***

# Supplementary Figures and Tables

## Supplementary Figures


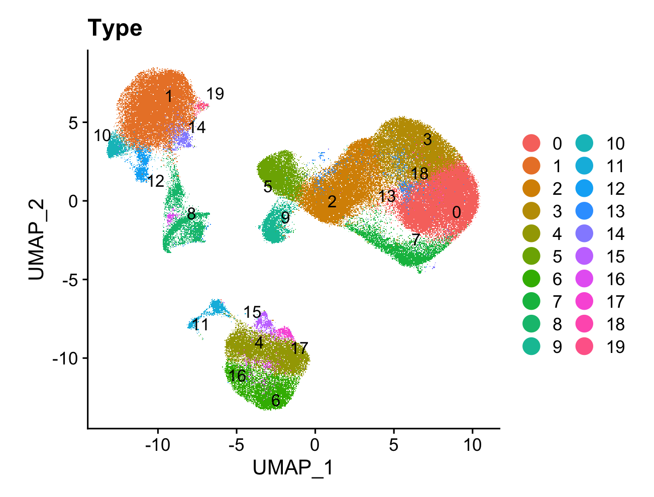


**Supplementary Figure 1.** A UMAP plot representing the 20 clusters across 140,785 PBMCs from 6 NMOSD patients before and after steroid therapies and 5 HCs.


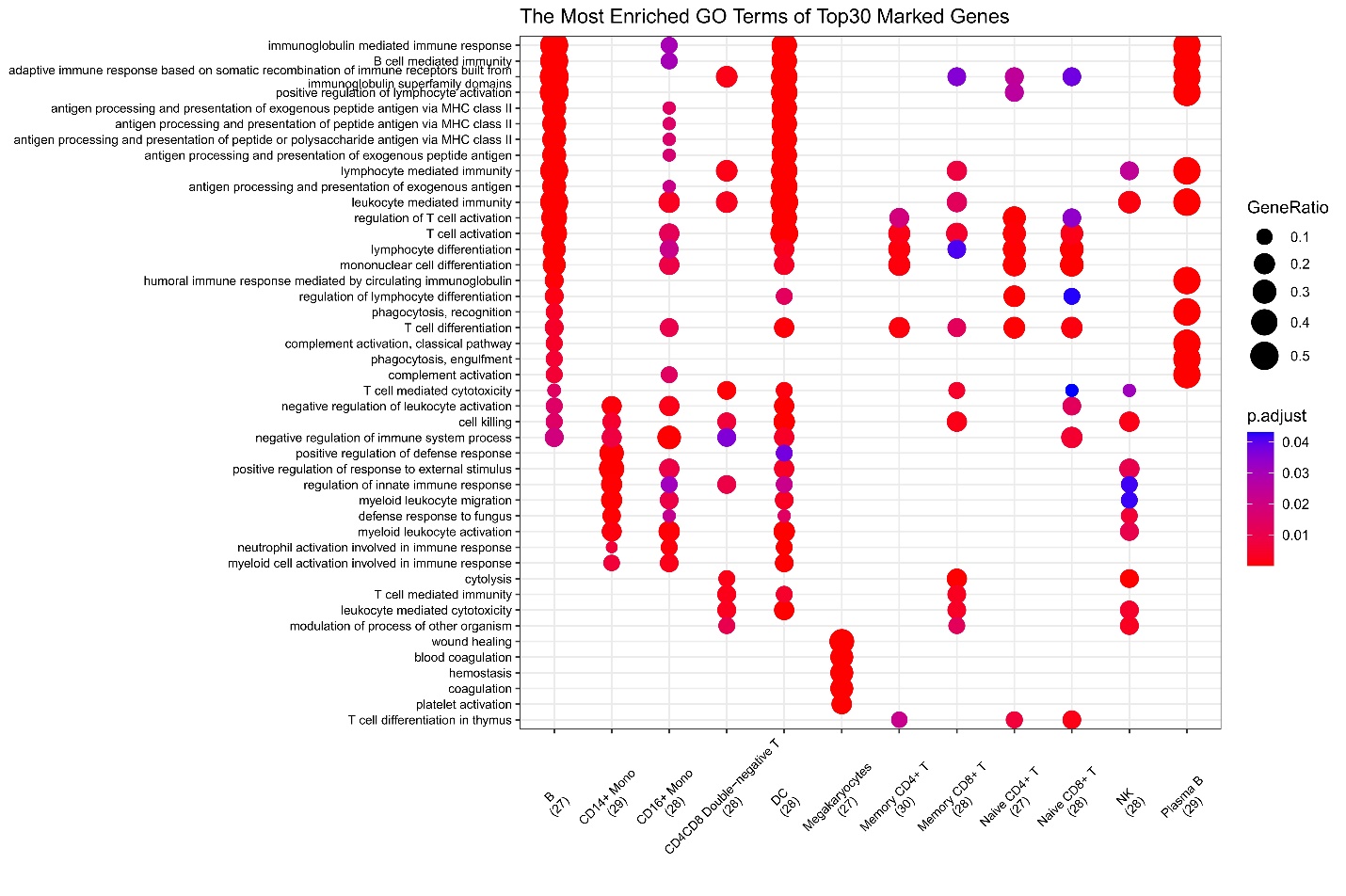


**Supplementary Figure 2.** GO enrichment analysis for major cell types.


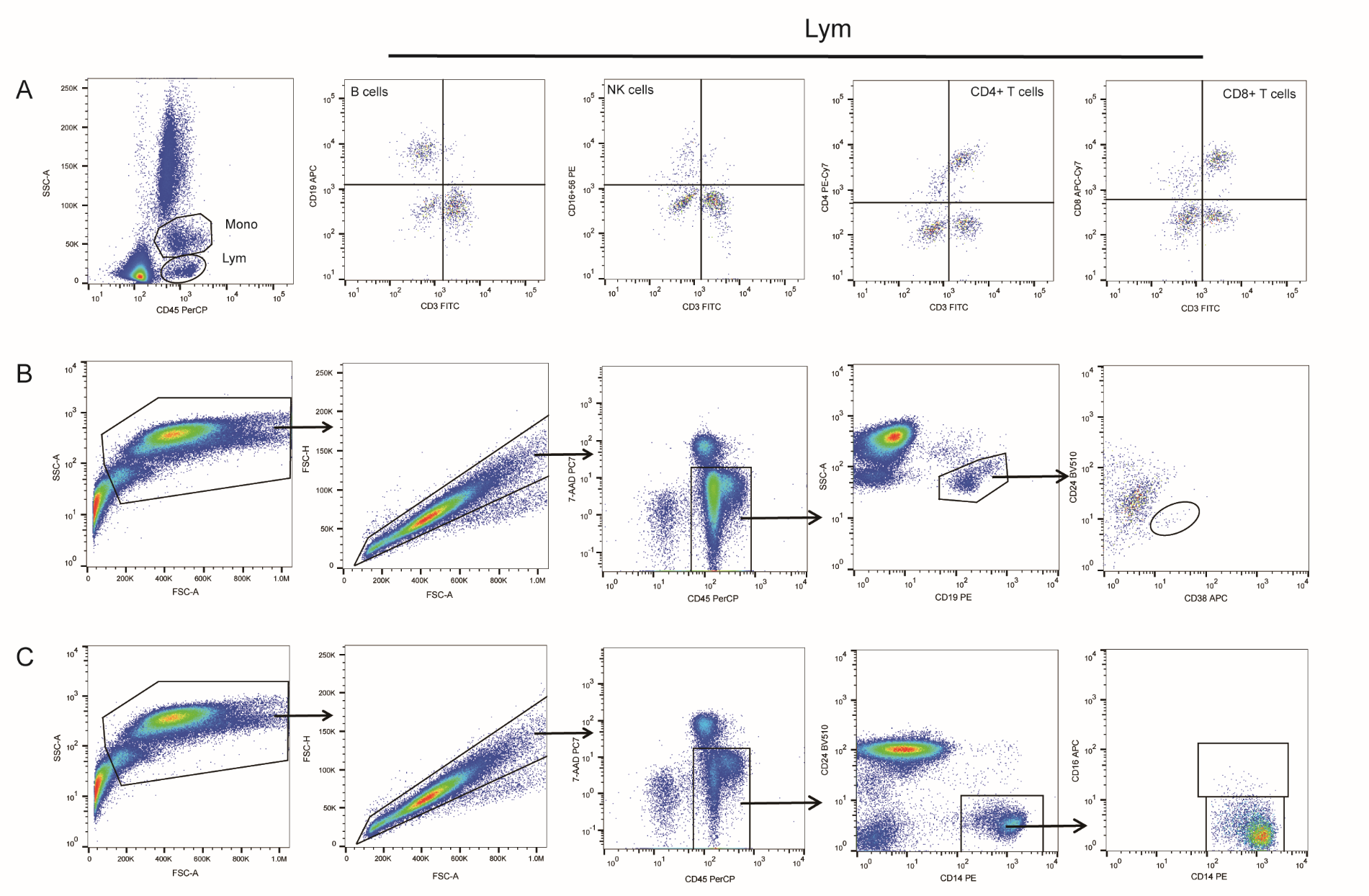


**Supplementary Figure 3.** Representative dot plots for flow cytometric analysis of peripheral blood monocytes and major lymphocyte populations (A), plasma B cells (B) and CD14+ monocytes (C).


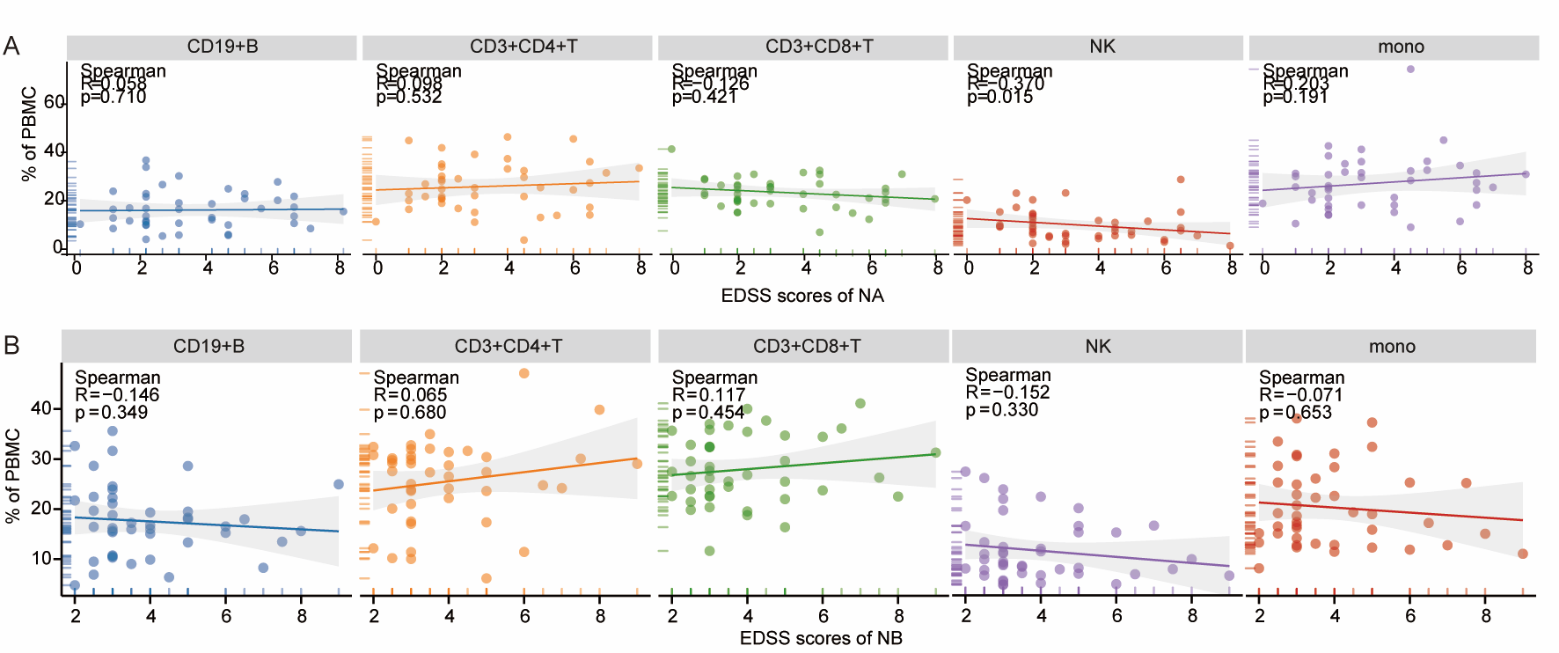


**Supplementary Figure 4.** Spearman’s correlation analysis between the EDSS scores and subtype representations under each condition.


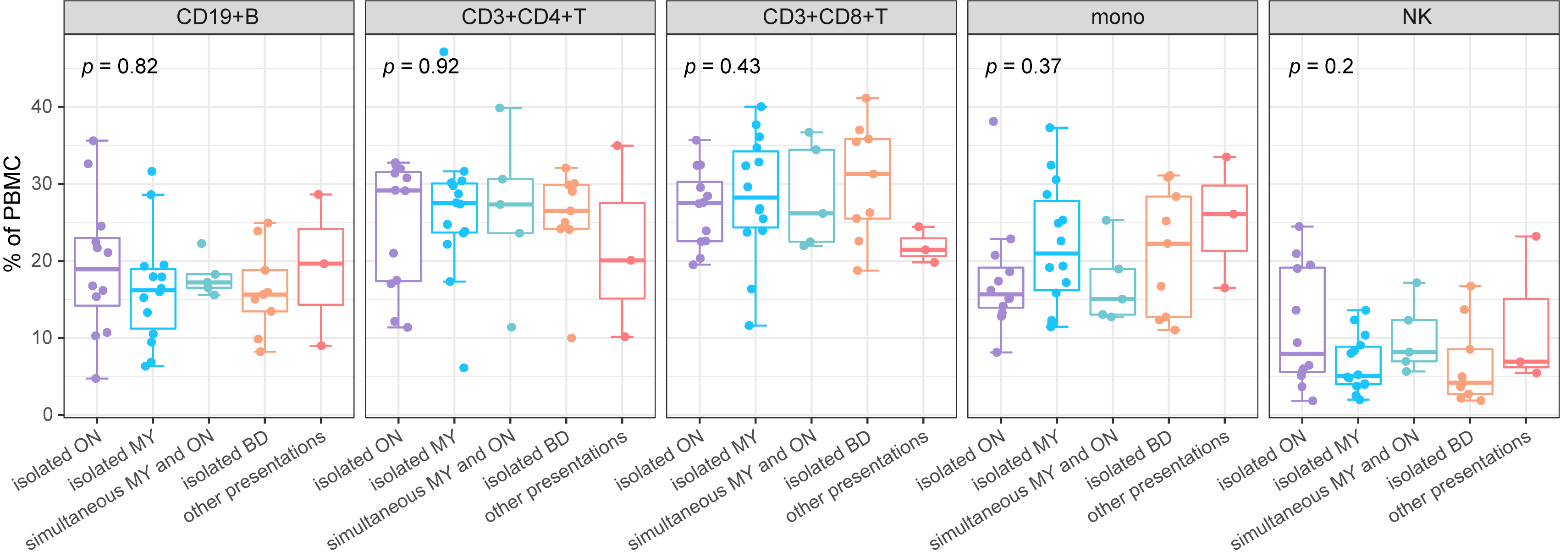


**Supplementary Figure 5.** Differences in each types of immune cell between the different attack manifestations of NMOSD patients.


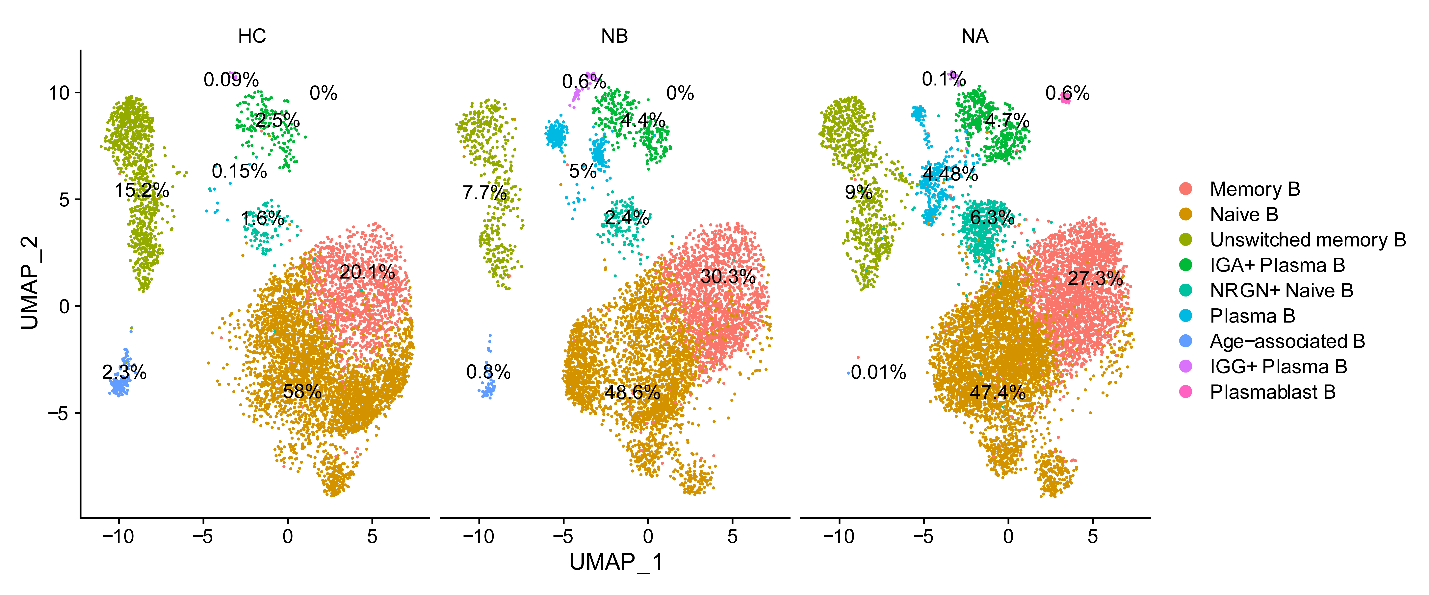


**Supplementary Figure 6.** UMAP projection of 9 B cell groups among health controls (n = 5) and patients (NB, n = 6; NA, n = 6).


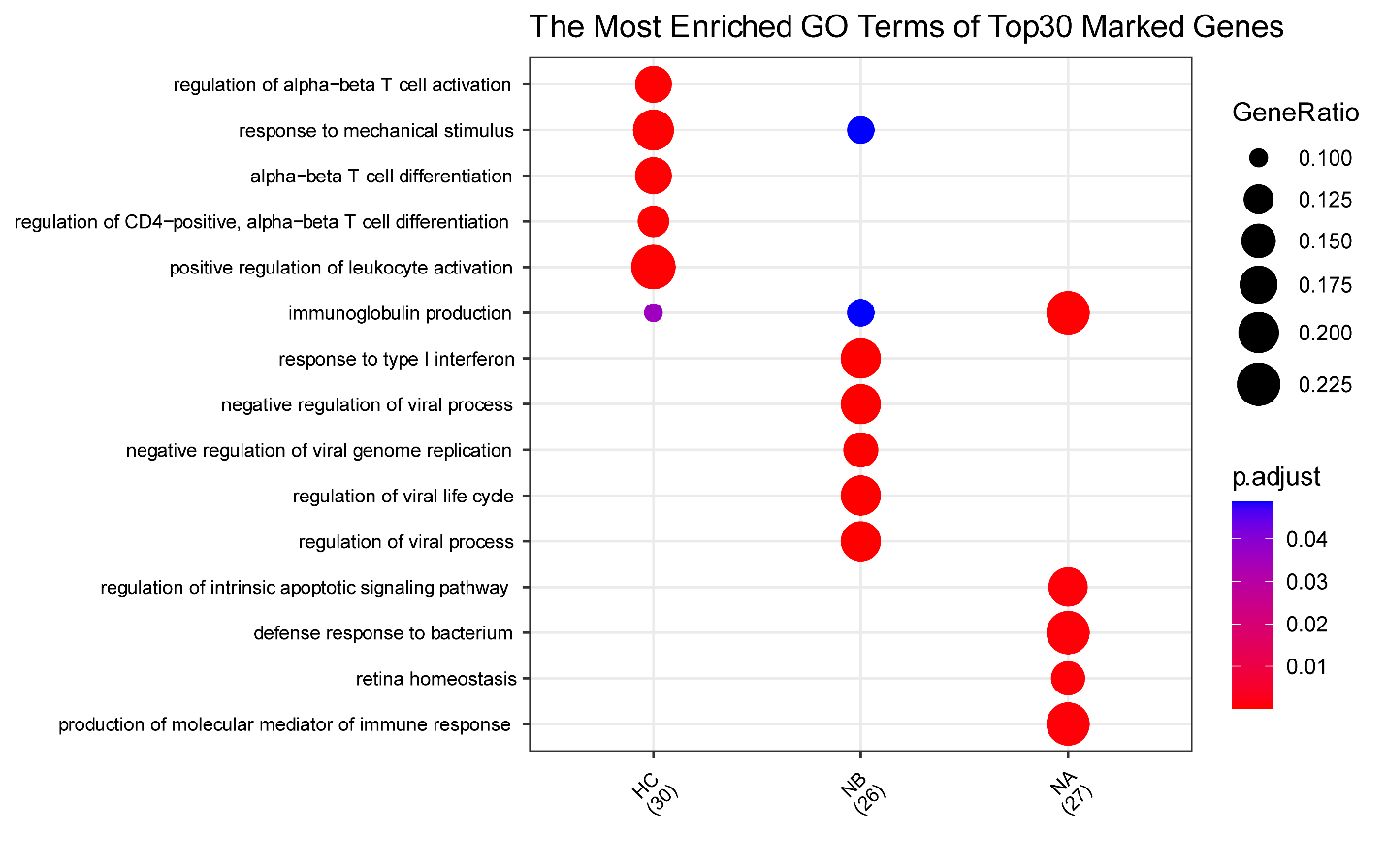


**Supplementary Figure 7.** Enrichment analysis of DEGs from B cells in NBs, NAs and HCs.


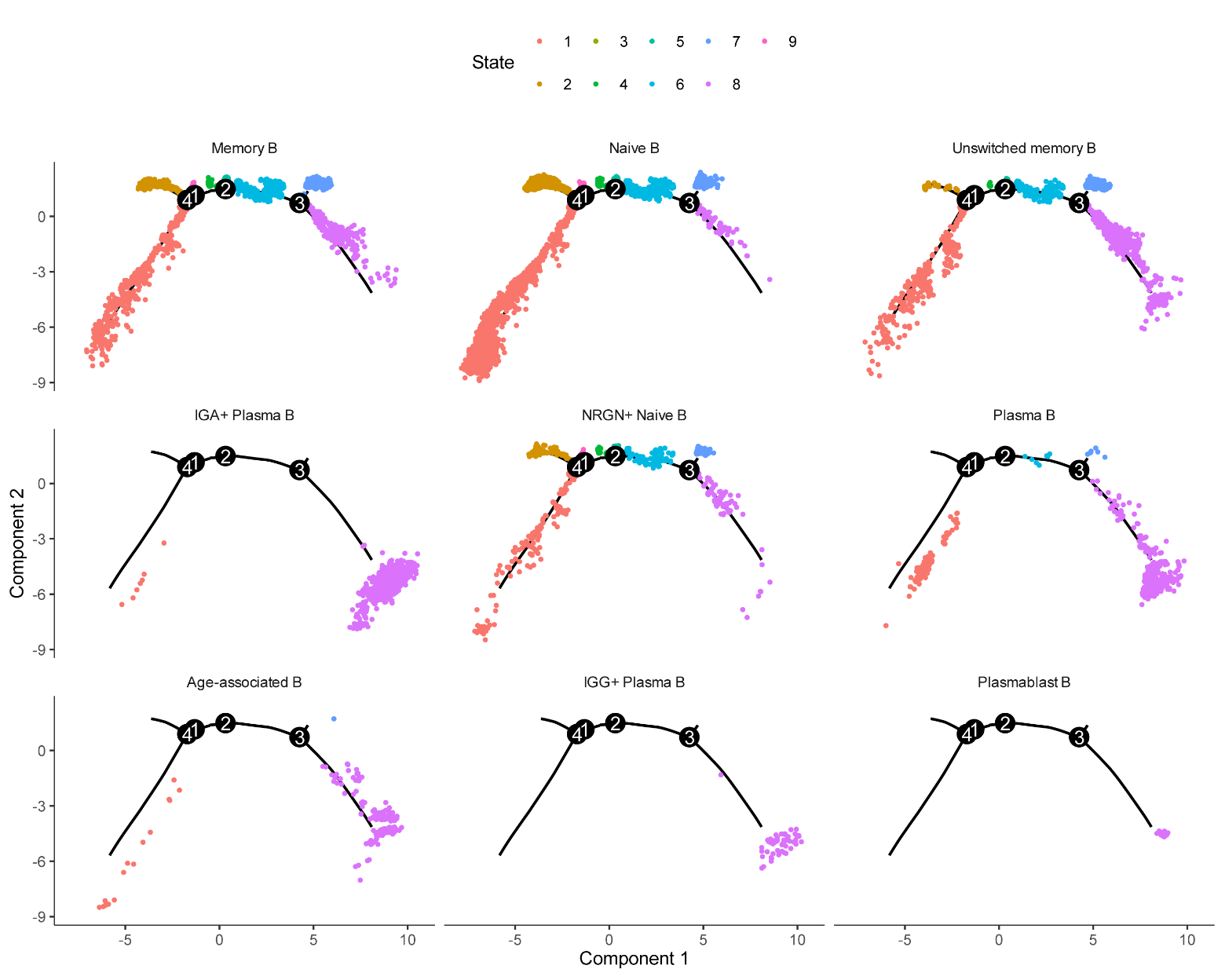


**Supplementary Figure 8.** Distribution of pseudotime among B cell subsets.


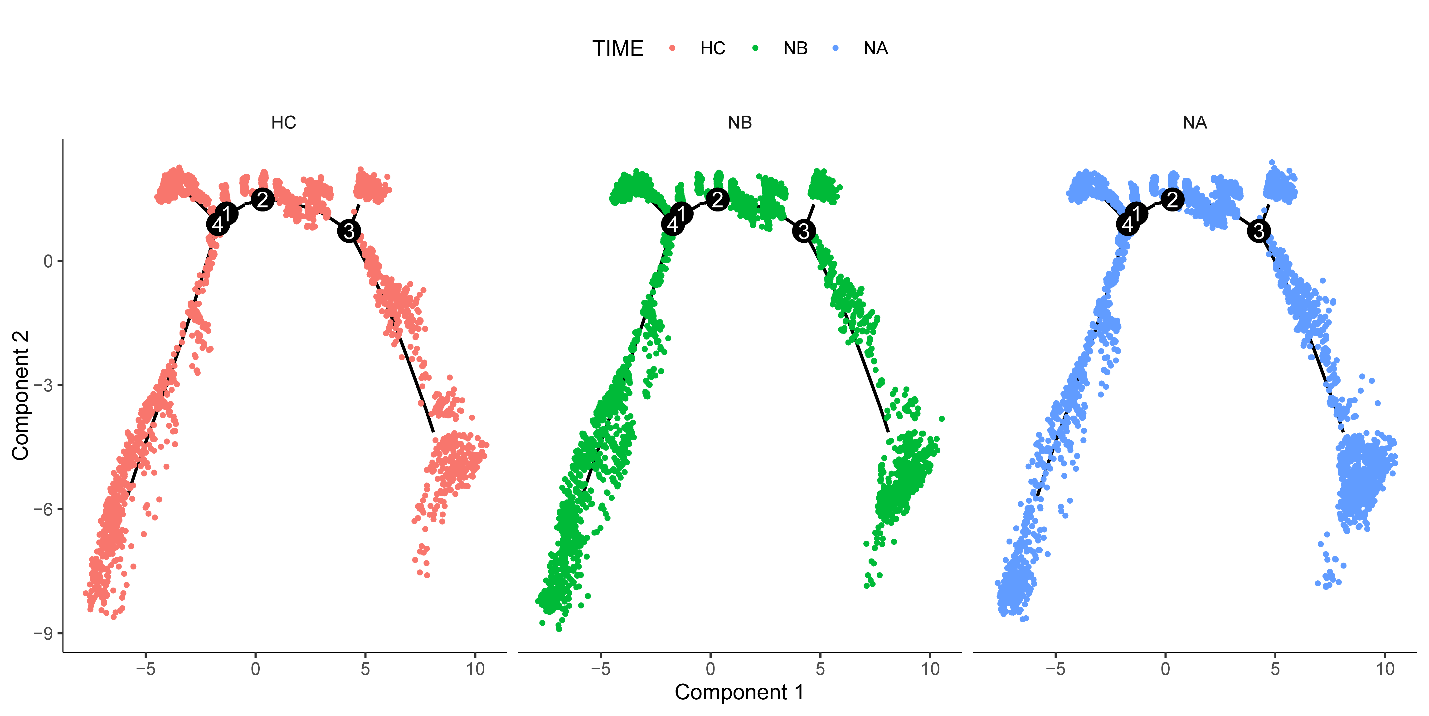


**Supplementary Figure 9.** Distribution of pseudotime among controls (n = 5) and patients (NB, n = 6; NA, n = 6).


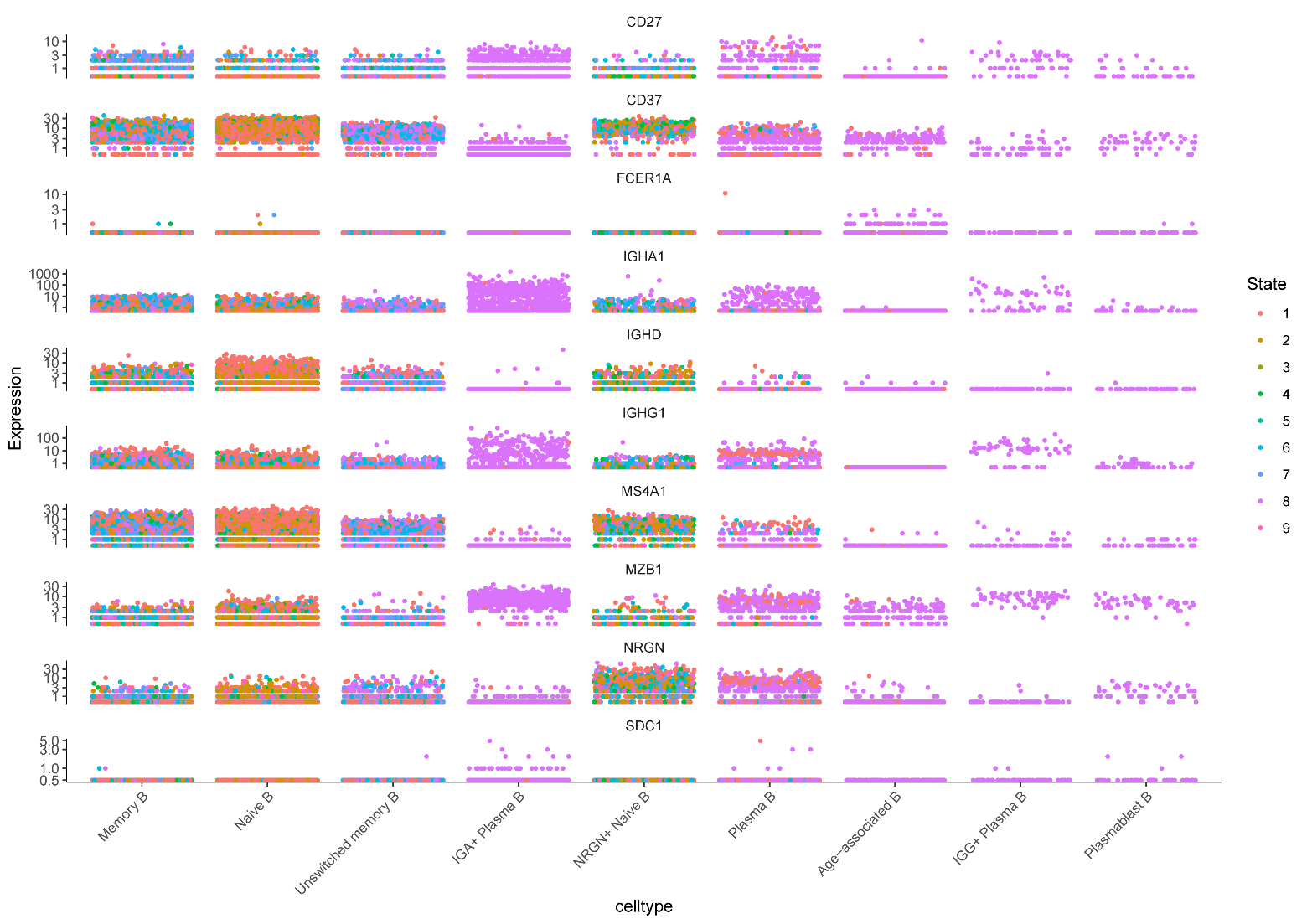


**Supplementary Figure 10**. Key genes related to differentiation in B cell subsets across each state.


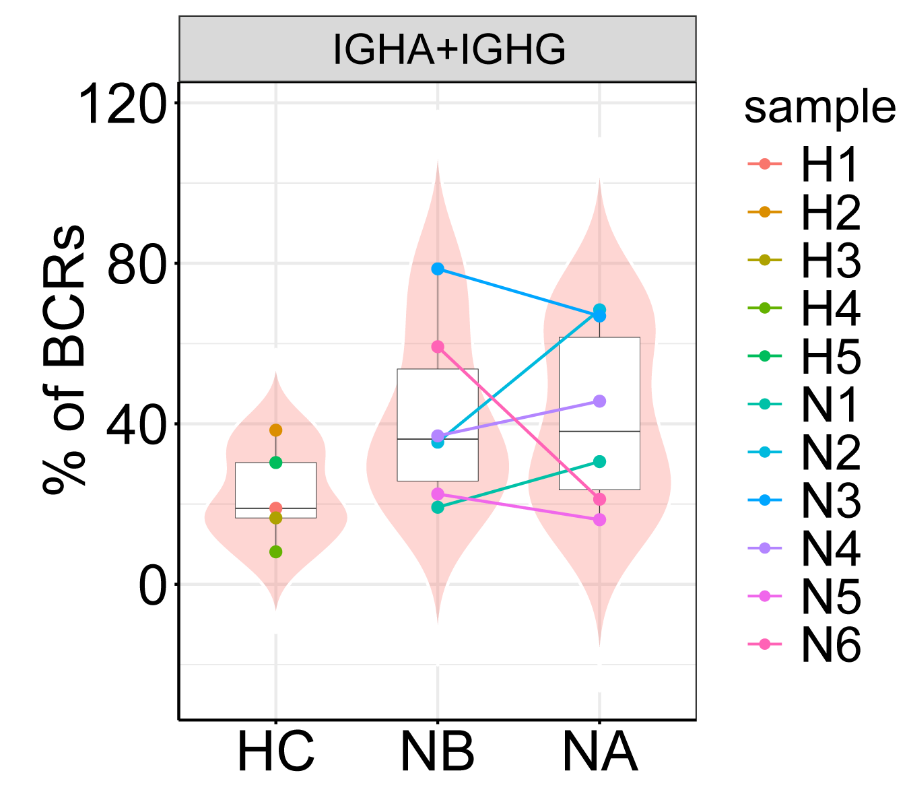


**Supplementary Figure 11.** Percentage of IGHA and IGHG in BCRs recovered by scBCR-seq among controls (n = 5) and patients (NB, n = 6; NA, n = 6). The samples are colored according to donors. *p*-values are calculated by using the Wilcoxon signed rank test.


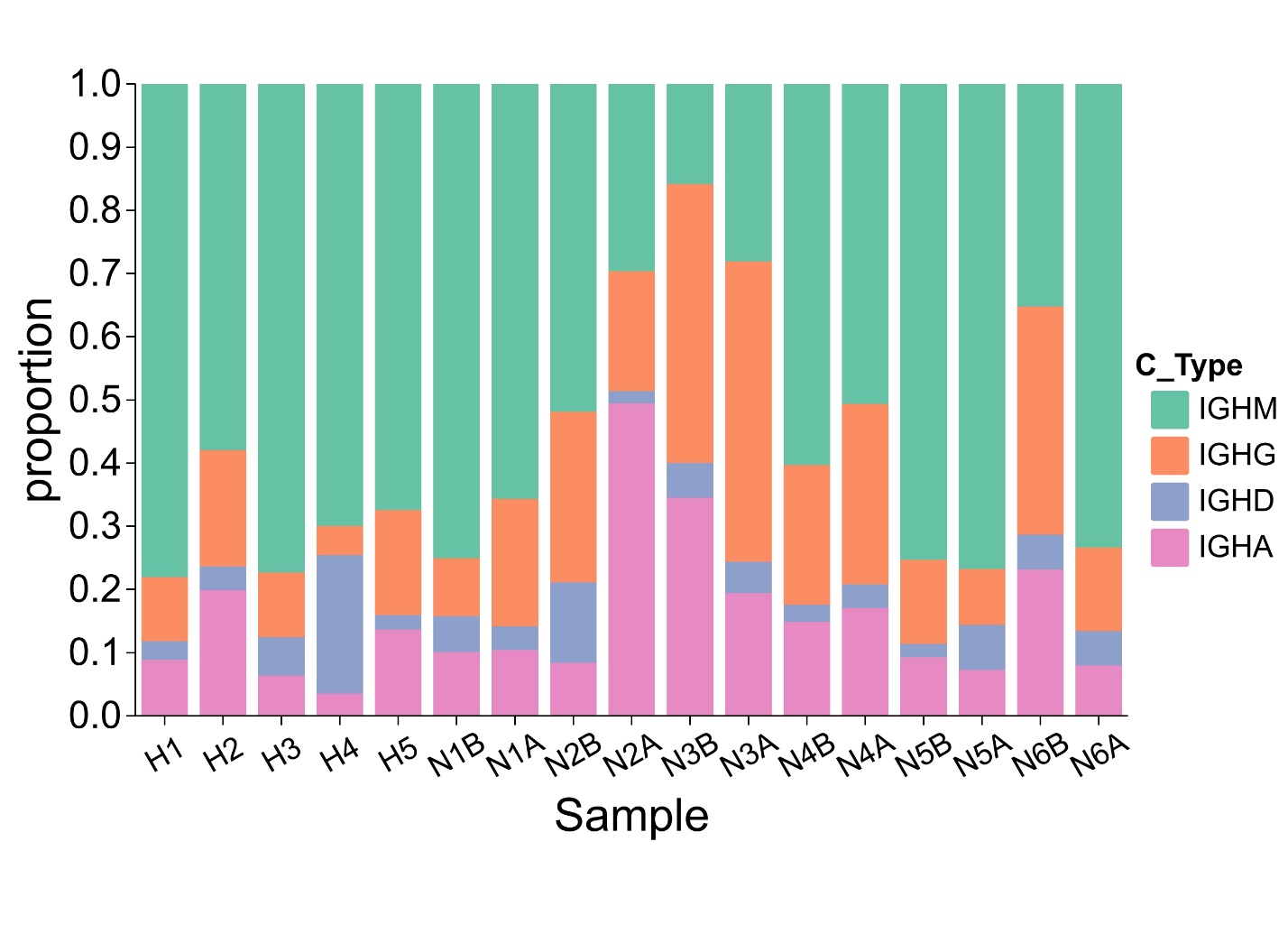


**Supplementary Figure 12.** Proportion of IGH isotypes in each controls and patients. Source data are provided in the Source Data file.


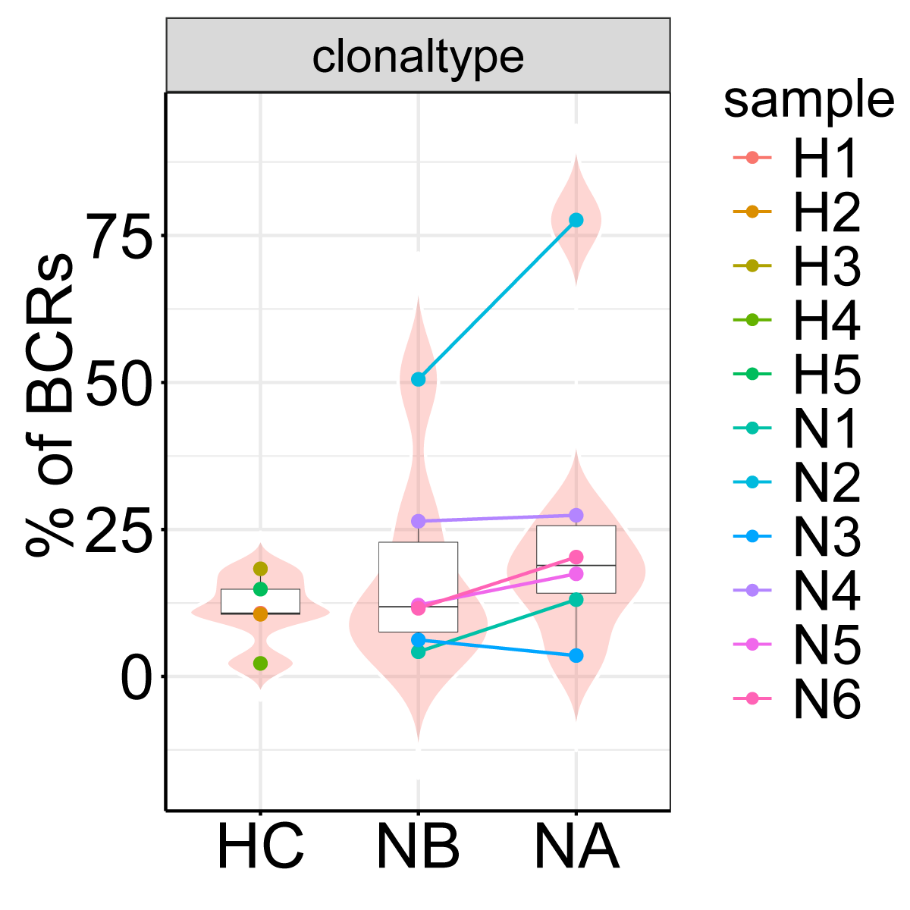


**Supplementary Figure 13.** Percentage of clonal BCR in BCRs recovered by scBCR-seq among controls (n = 5) and patients (NB, n = 6; NA, n = 6). The samples are colored according to donors. *p*-values are calculated by using the Wilcoxon signed rank test.


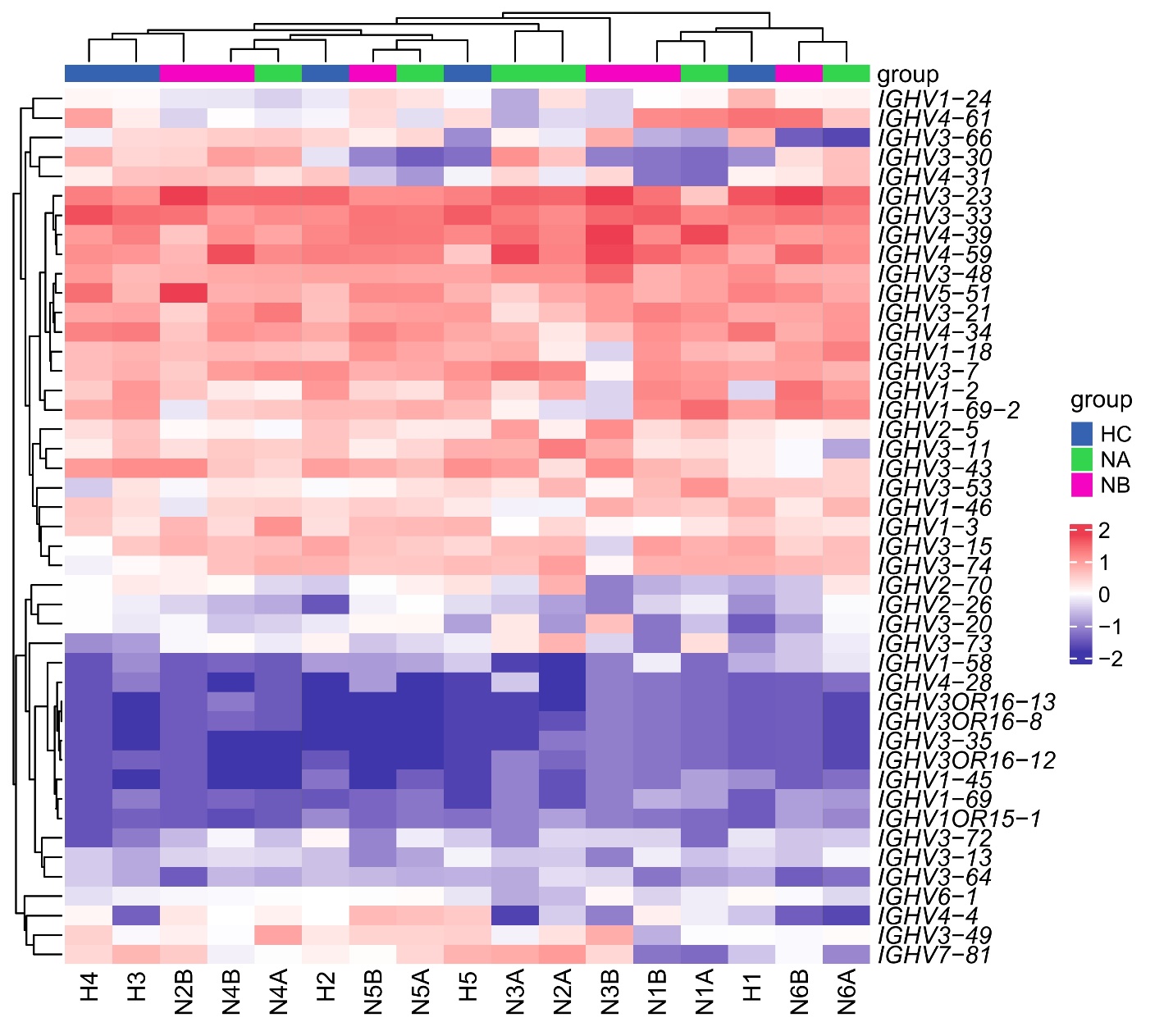


**Supplementary Figure 14.** IGHV gene usage in the BCR repertoires. Heat map represents proportion of IGHV genes across samples. Source data are provided in the Source Data file.


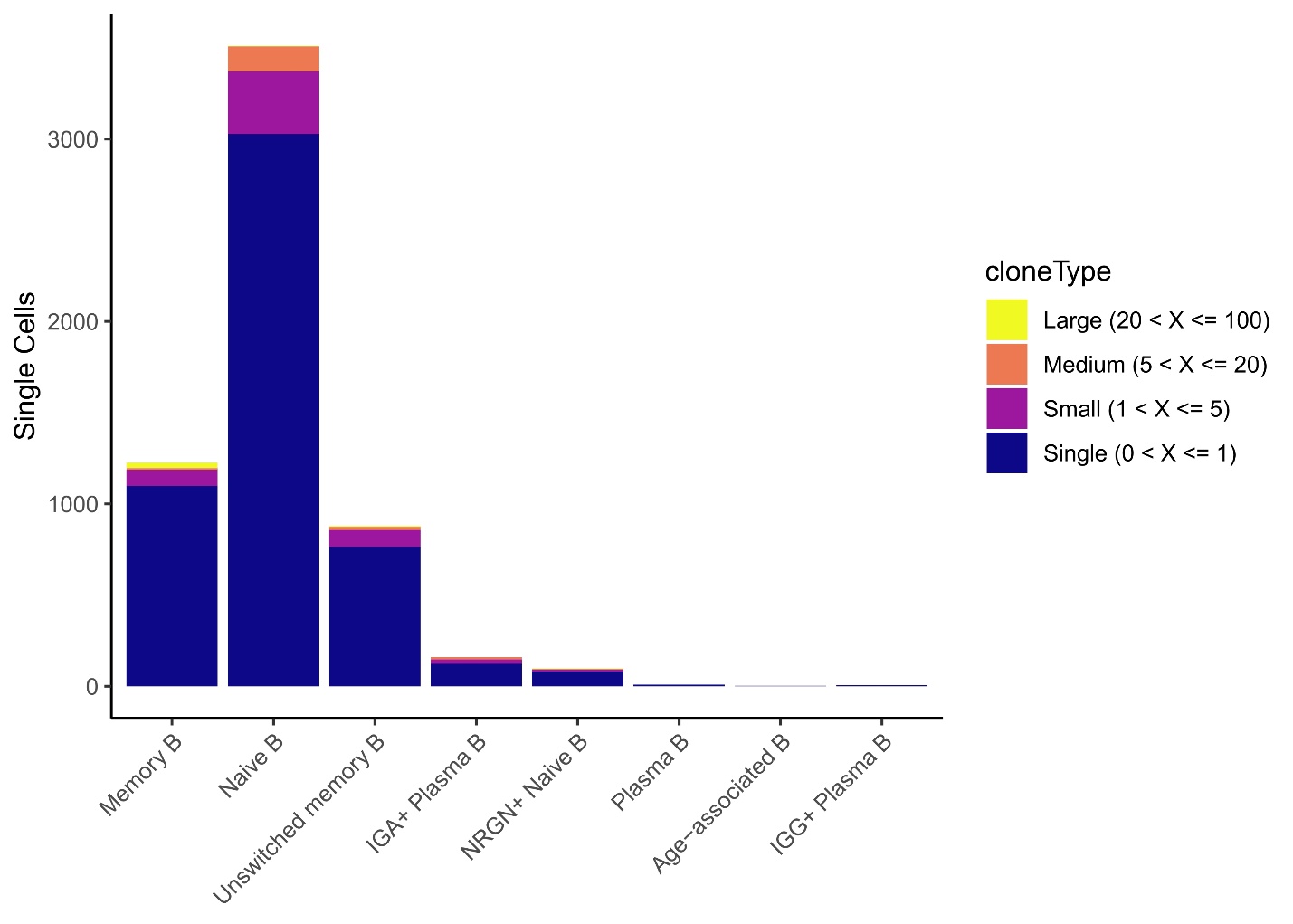


**Supplementary Figure 15.** BCR clonotype tracking in B cell subsets.


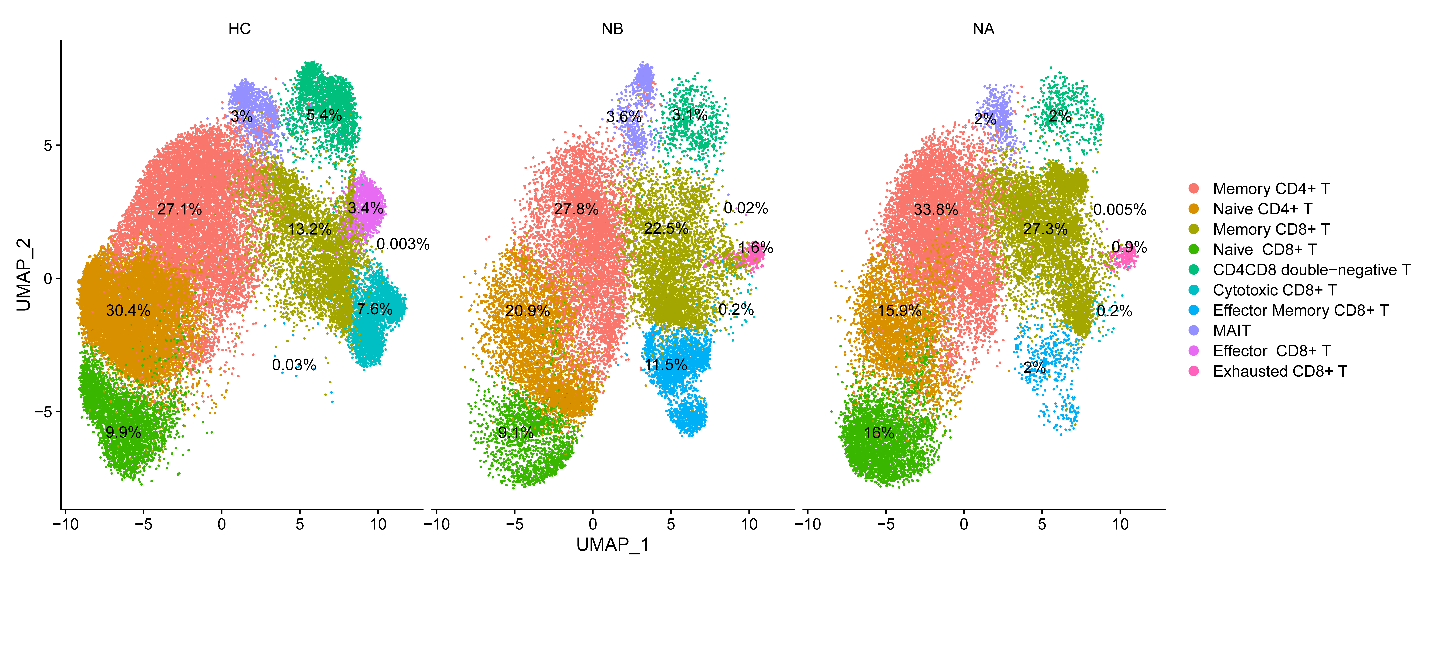


**Supplementary Figure 16.** UMAP projection of 10 T cell groups among health controls (n = 5) and patients (NB, n = 6; NA, n = 6).


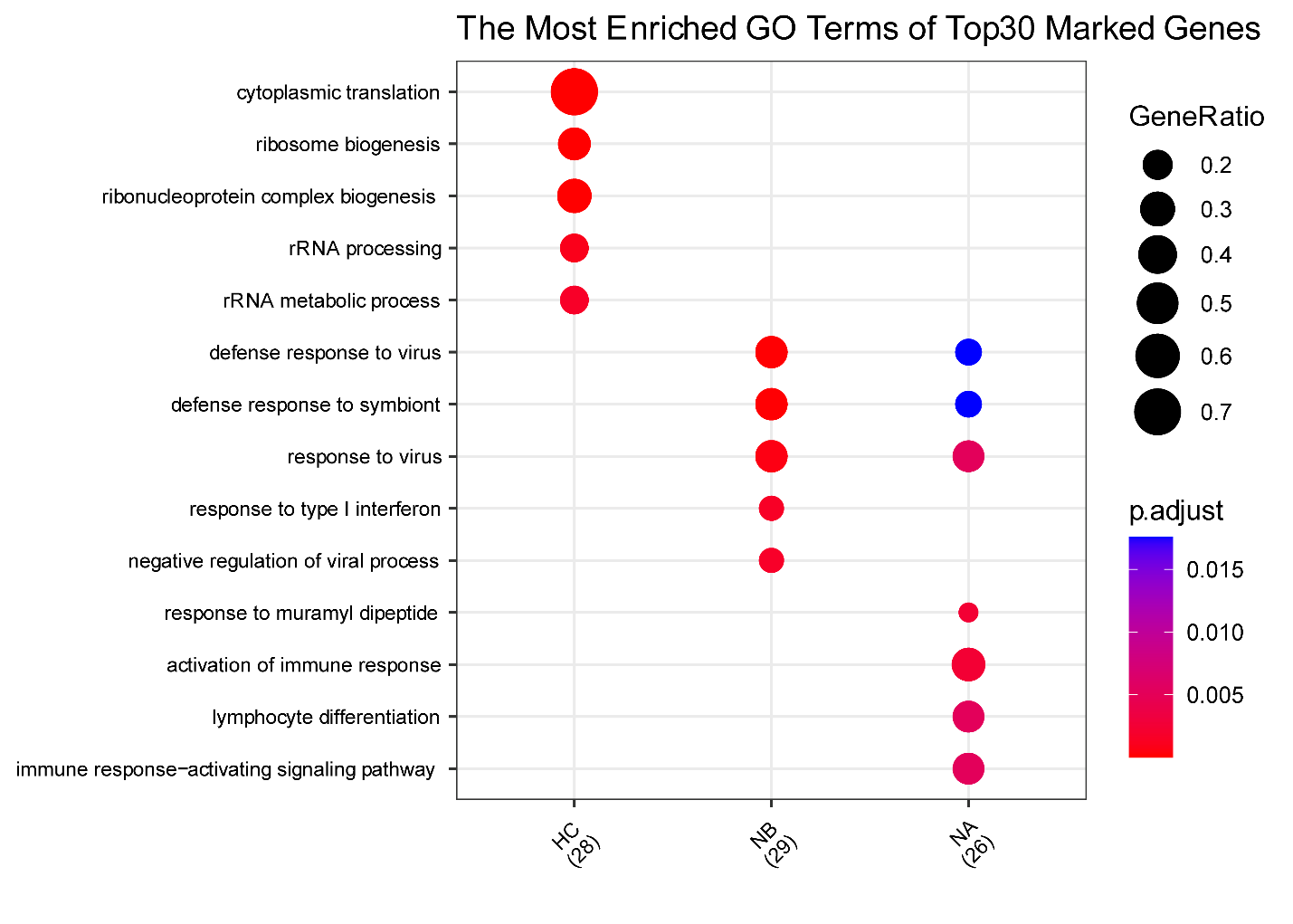


**Supplementary Figure 17.** GO enrichment analysis of DEGs from T cells in NBs, NAs and HCs.


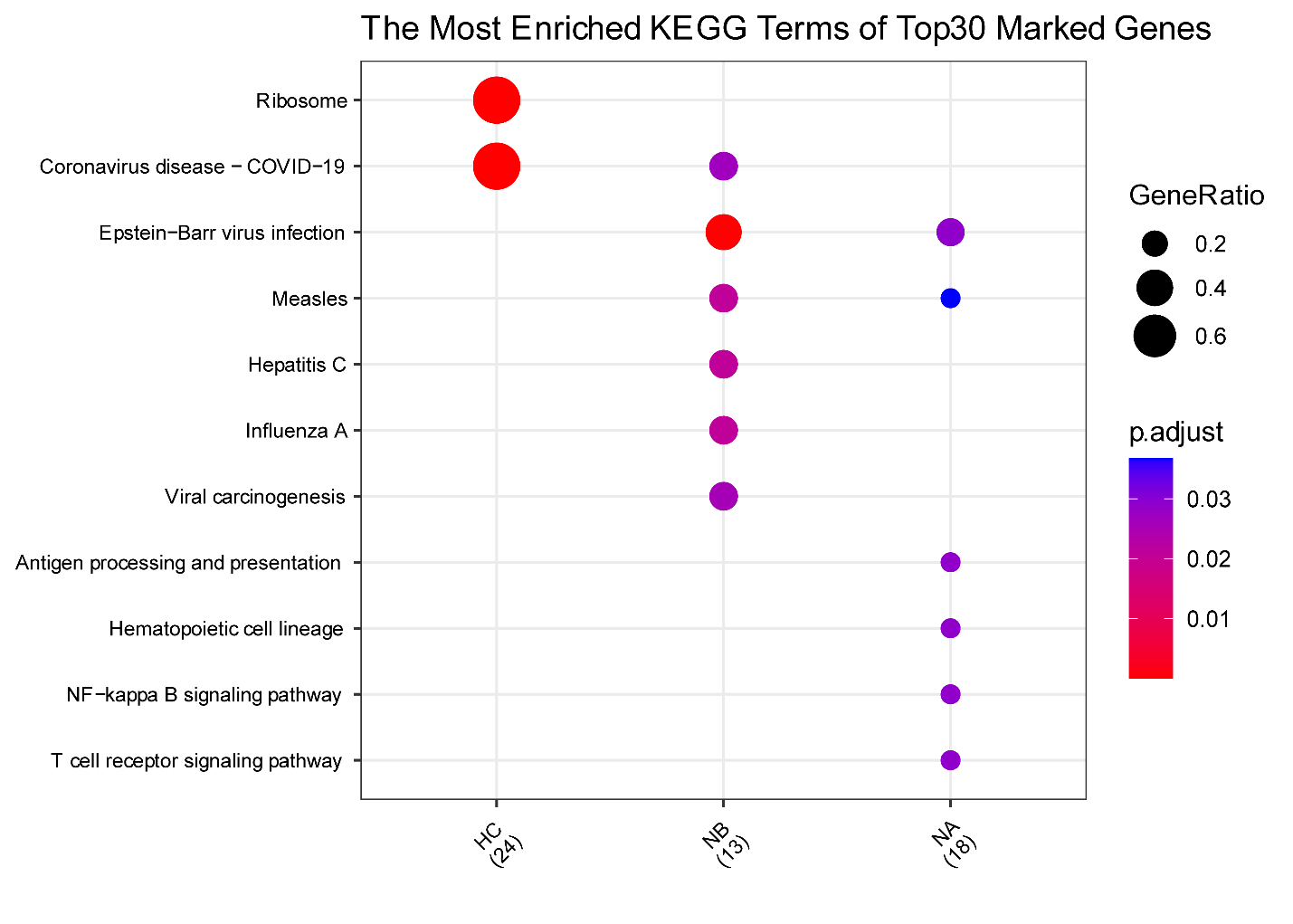


**Supplementary Figure 18.** KEGG enrichment analysis of DEGs from T cells in NBs, NAs and HCs.


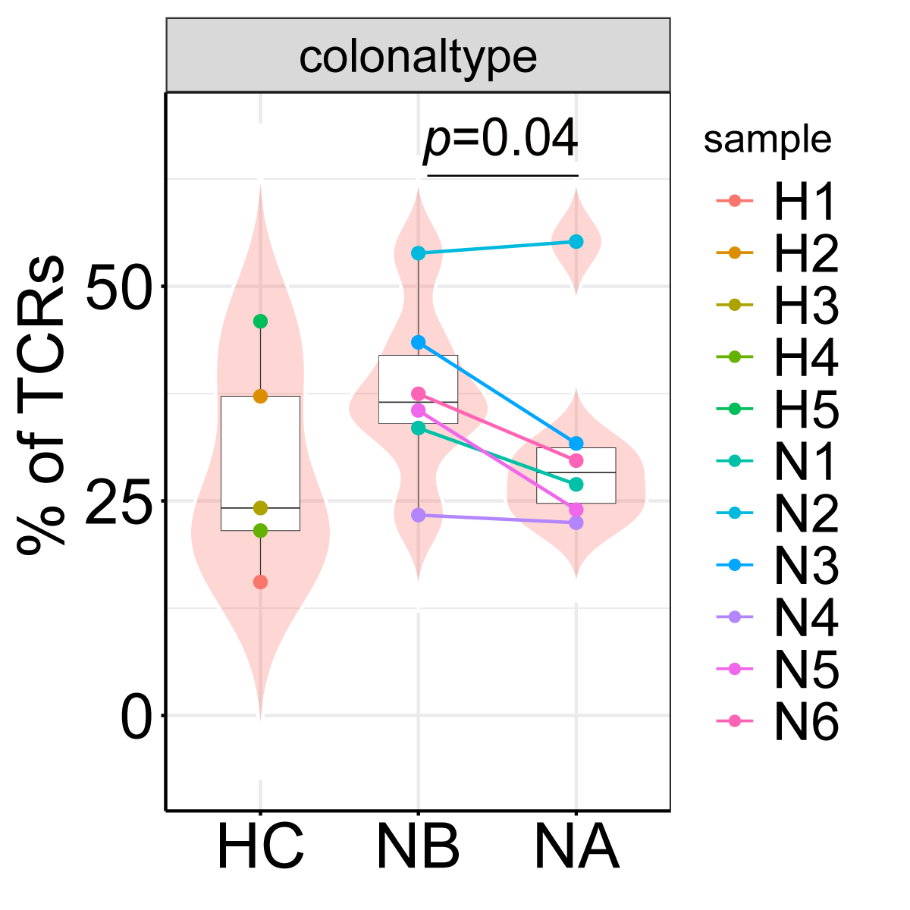


**Supplementary Figure 19.** Percentage of clonal TCR in TCRs recovered by scBCR-seq among controls (n = 5) and patients (NB, n = 6; NA, n = 6). The samples are colored according to donors. *p*-values are calculated by using the Wilcoxon signed rank test.


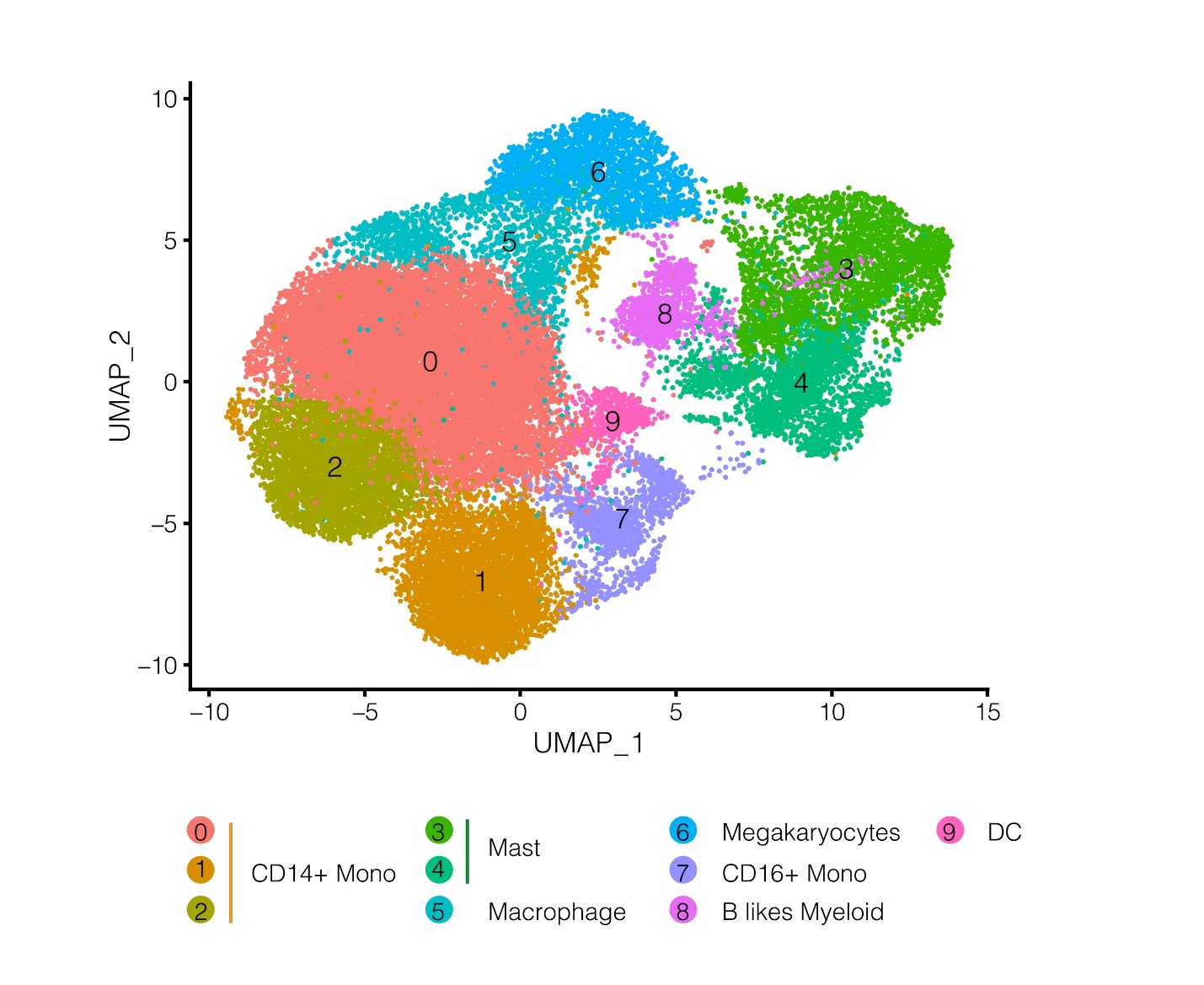


**Supplementary Figure 20.** The heterogeneous clusters of myeloid cells were shown by UMAP.


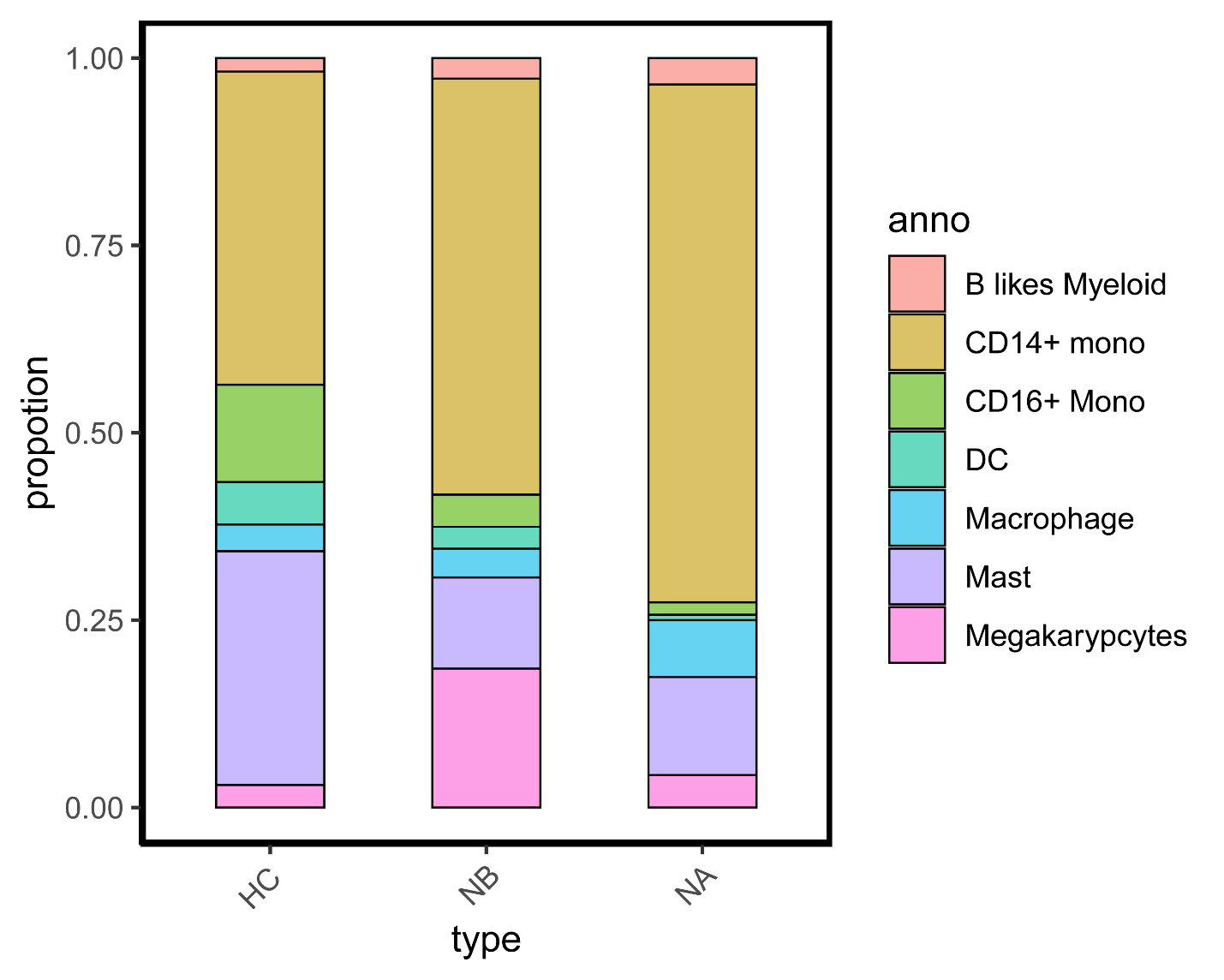


**Supplementary Figure 21.** Percentage of myeloid cell types under each condition.


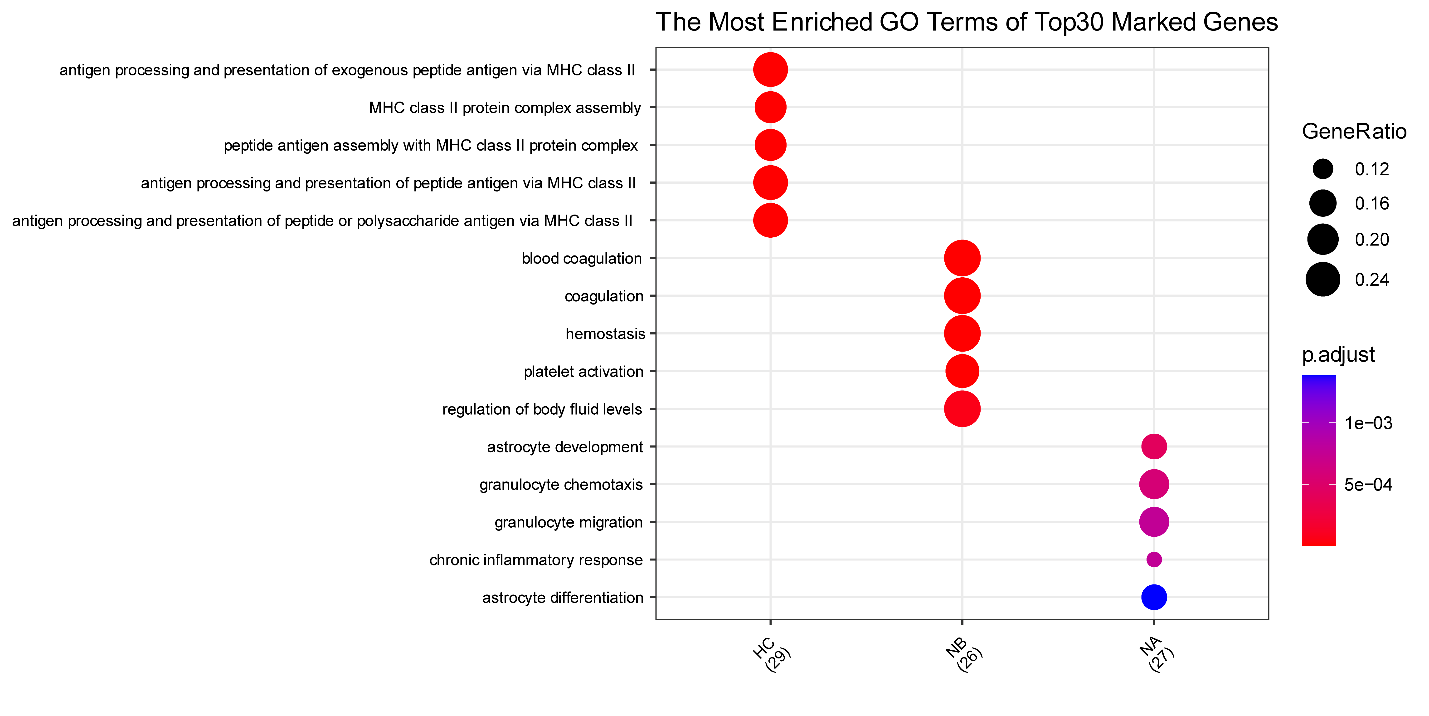


**Supplementary Figure 22.** GO enrichment analysis of DEGs from myeloid cells in NBs, NAs and HCs


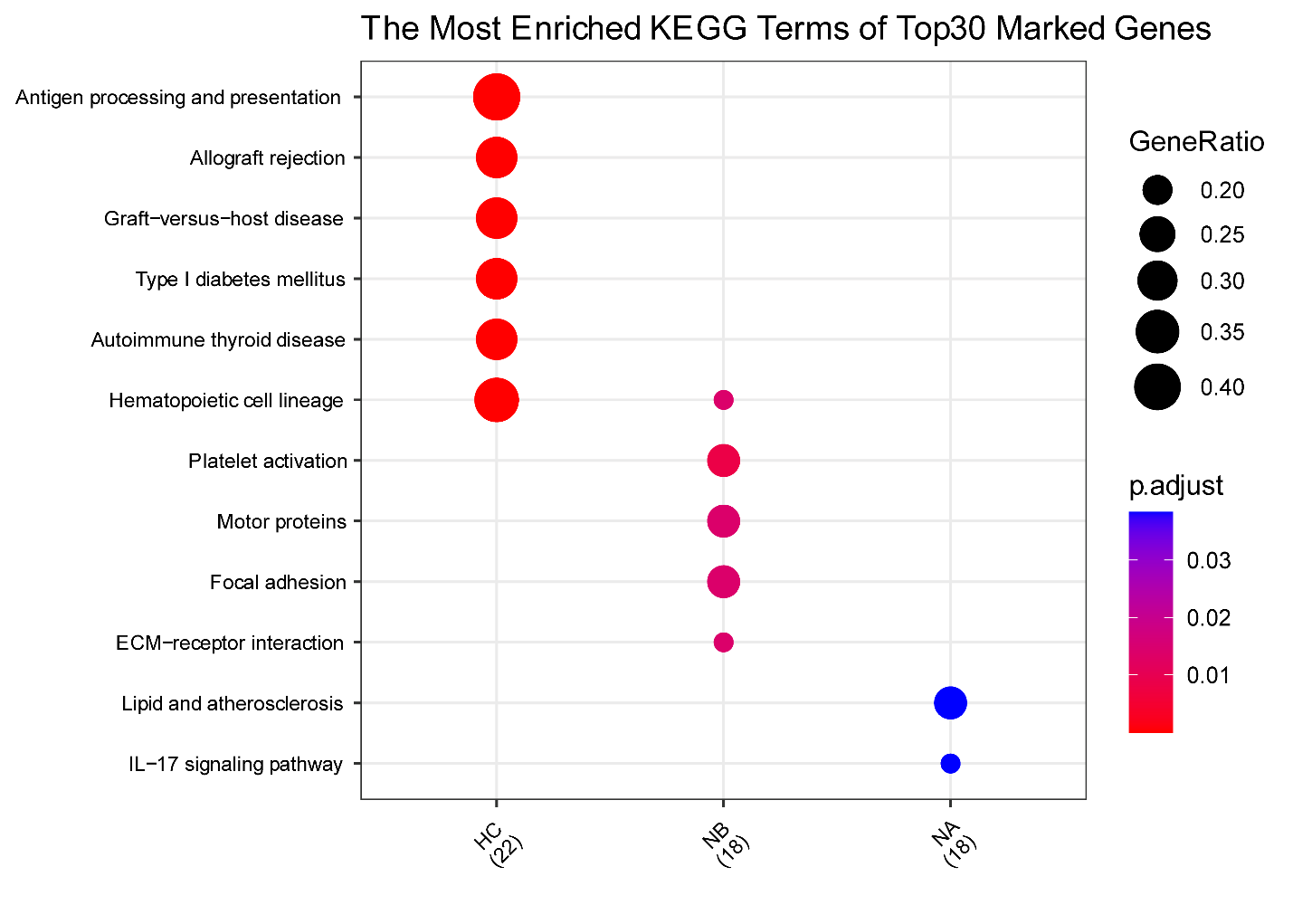


**Supplementary Figure 23.** GO enrichment analysis of DEGs from myeloid cells in NBs, NAs and HCs.


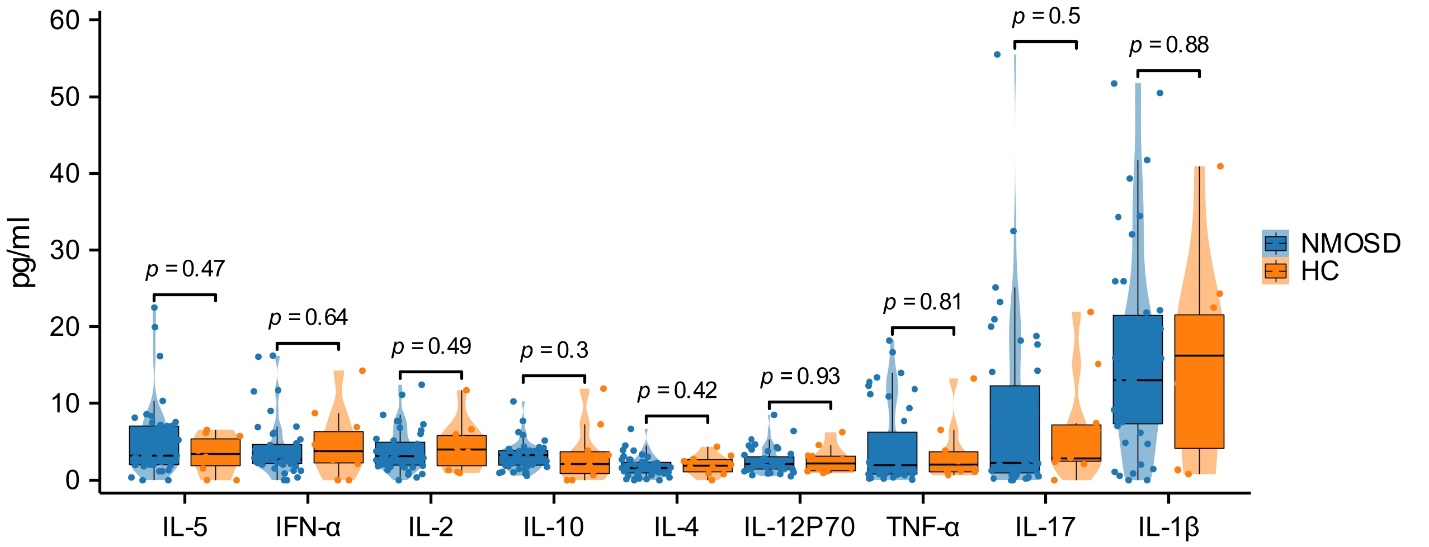


**Supplementary Figure 24.** The levels of selected cytokines and chemokines in peripheral blood across HCs (n = 10), PBs (n = 43).


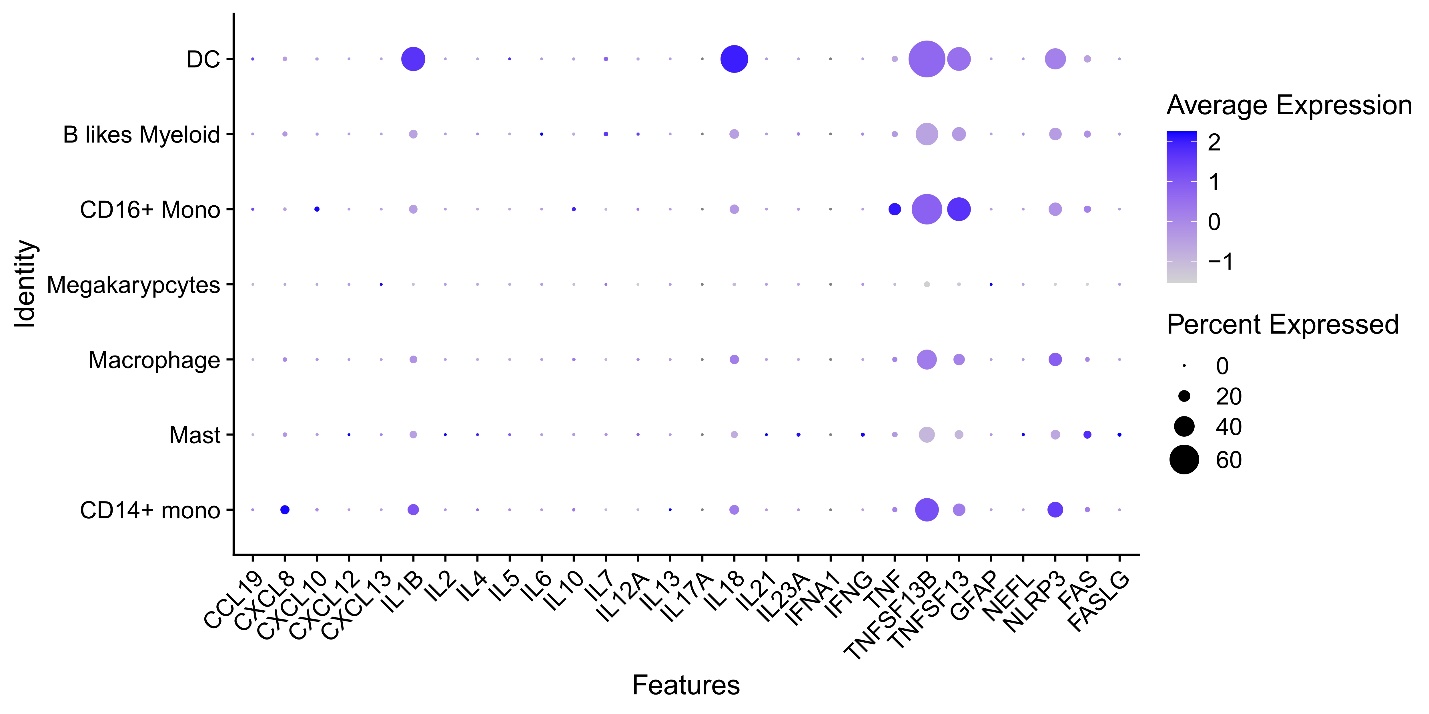


**Supplementary Figure 25.** Expression patterns of molecular biomarkers in myeloid cells.

## Supplementary Tables

**Supplementary table 1. Demographic characteristics of study populations**

| **Category** | **Cohort 1** | | **Cohort 2** | |
| --- | --- | --- | --- | --- |
|  | **NMOSD(n=6)** | **HC(n=5)** | **NMOSD(n=43)** | **HC(n=10)** |
| Age, mean (SD) | 32.50±16.65 | 24.50±6.40 | 42.372±20.39 | 34.4±12.17 |
| Female, n (%) | 6(100) | 3(60) | 33(76.74) | 5(50) |
| EDSS at start of steroid therapy (median, IQR) | 4(2.88-5.75) | - | 3(3-5) | - |
| EDSS at 10 days after the initiation of steroid therapy (median, IQR) | 4.25(2.63-6.63) | - | 3(2-5) | - |
| Characteristics of attacks |  |  |  |  |
| isolated MY | 2(33.333%) | - | 14(32.558%) | - |
| isolated ON | 2(33.333%) | - | 12(27.907%) | - |
| isolated BD | 1(16.667%) | - | 9(20.93%) | - |
| simultaneous MY and ON | 1(16.667%) | - | 5(11.628%) | - |
| other presentations | 0 | - | 3(6.977%) | - |
| Movement disorders, n (%) | 2(33.33%) | 0 | 26(60.47%) | 0 |
| sensory disorders, n (%) | 3(50%) | 0 | 22(51.16%) | 0 |
| decreased vision, n (%) | 3(50%) | 0 | 20(46.51%) | 0 |
| nausea, n (%) | 1(16.67%) | 0 | 6(13.95%) | 0 |
| vomiting, n (%) | 1(16.67%) | 0 | 6(13.95%) | 0 |
| dizziness, n (%) | 1(16.67%) | 0 | 4(9.3%) | 0 |
| Hiccups, n (%) | 1(16.67%) | 0 | 4(9.3%) | 0 |
| cough, n (%) | 1(16.67%) | 0 | 3(6.98%) | 0 |
| diplopia, n (%) | 1(16.67%) | 0 | 2(4.65%) | 0 |
| Urinary and fecal disorders, n (%) | 0 | 0 | 2(4.65%) | 0 |
| drowsiness, n (%) | 0 | 0 | 2(4.65%) | 0 |
| memory loss, n (%) | 0 | 0 | 1(2.33%) | 0 |
| headache, n (%) | 1(16.67%) | 0 | 1(2.33%) | 0 |
| speech impairment, n (%) | 1(16.67%) | 0 | 1(2.33%) | 0 |
| nystagmus, n (%) | 0 | 0 | 1(2.33%) | 0 |

NMOSD, Neuromyelitis optica spectrum disorder; HC, healthy control; EDSS: Expanded Disability Status Scale; MY, myelitis; ON, optic neuritis; BD, brainstem dysfunction

**Supplementary table 2. Summary of single-cell RNA sequencing data**

| **Sample** | **Cell Count.Raw** | **Cell Count.Filtered** | **Median UMI Count.Raw** | **Median UMI Count.Filtered** | **Median Gene Count.Raw** | **Median Gene Count.Filtered** | **Median Percent MT.Raw(%)** | **Median Percent MT.filtered(%)** |
| --- | --- | --- | --- | --- | --- | --- | --- | --- |
| HC1 | 12518 | 12301 | 3976 | 4001 | 1474 | 1481 | 2.58524976 | 2.56293944 |
| HC2 | 14161 | 14146 | 3658 | 3658 | 1408 | 1408 | 2.83687943 | 2.83588844 |
| HC3 | 9294 | 8730 | 3564 | 3675.5 | 1342 | 1370 | 2.19538911 | 2.11141238 |
| HC4 | 5748 | 3888 | 1748.5 | 2374 | 891.5 | 1100.5 | 6.4921377 | 4.38796569 |
| HC5 | 14071 | 14060 | 3413 | 3413 | 1420 | 1421 | 1.38053097 | 1.38055158 |
| N1a | 6076 | 5868 | 2987 | 3078 | 1337 | 1366 | 2.49208011 | 2.42697228 |
| N1b | 2724 | 2620 | 3152 | 3231.5 | 1433 | 1464 | 2.07914307 | 2.01214882 |
| N2a | 8761 | 7892 | 2586 | 2714 | 1262 | 1305 | 4.85496183 | 4.62065756 |
| N2b | 3101 | 2533 | 1702 | 2311 | 852 | 1111 | 5.17458982 | 4.72111175 |
| N3a | 5810 | 5245 | 3021.5 | 3199 | 1257 | 1315 | 2.60257607 | 2.38945345 |
| N3b | 2293 | 1479 | 1617 | 2328 | 787 | 1071 | 5.82608696 | 4.03616403 |
| N4a | 10577 | 10280 | 3163 | 3208 | 1288 | 1301 | 2.25856698 | 2.20540126 |
| N4b | 11789 | 11467 | 3698 | 3746 | 1488 | 1500 | 2.09504343 | 2.05574913 |
| N5a | 12808 | 12389 | 3642 | 3697 | 1460 | 1475 | 2.44464285 | 2.41234222 |
| N5b | 10545 | 10152 | 3842 | 3901 | 1604 | 1623 | 1.8940248 | 1.86490998 |
| N6a | 13436 | 12942 | 3702 | 3766.5 | 1414 | 1432 | 3.30036675 | 3.24091635 |
| N6b | 5711 | 4793 | 1576 | 1784 | 864 | 937 | 4.62794918 | 3.92596747 |

b, before; a, after;

**Supplementary table 3. Summary of single-cell BCR sequencing data**

| **Samples** | **Number of Cells** | **Mean Read Pairs per Cell** | **Number of cells with clonotypes** | **Number of clonotypes** | **Number of cells with productive paired chains** |
| --- | --- | --- | --- | --- | --- |
| HC1 | 808 | 53,650 | 808 | 808 | 551 |
| HC2 | 1501 | 23169 | 1501 | 1,485 | 1,039 |
| HC3 | 2364 | 14883 | 2364 | 2,331 | 1,518 |
| HC4 | 616 | 56,000 | 616 | 551 | 469 |
| HC5 | 1,401 | 28,609 | 1,401 | 1327 | 960 |
| N1b | 410 | 102,101 | 410 | 399 | 277 |
| N1a | 591 | 76,740 | 591 | 551 | 387 |
| N2b | 773 | 123,967 | 773 | 336 | 472 |
| N2a | 6,724 | 12,745 | 6,724 | 1,679 | 4,290 |
| N3b | 183 | 176,540 | 183 | 170 | 104 |
| N3a | 516 | 75,047 | 516 | 511 | 328 |
| N4b | 3,070 | 11,786 | 3,070 | 2,780 | 2,198 |
| N4a | 1,945 | 18,889 | 1,945 | 1651 | 1,337 |
| N5b | 1,340 | 33,009 | 1,340 | 1,302 | 951 |
| N5a | 2,486 | 14,569 | 2,486 | 2,412 | 1,754 |
| N6b | 434 | 78,342 | 434 | 405 | 265 |
| N6a | 2,686 | 18,113 | 2,686 | 2,528 | 2,008 |

b, before; a, after;

**Supplementary table 4. Summary of single-cell TCR sequencing data**

| **Samples** | **Number of Cells** | **Mean Read Pairs per Cell** | **Number of cells with clonotypes** | **Number of clonotypes** | **Number of cells with productive paired chains** |
| --- | --- | --- | --- | --- | --- |
| HC1 | 7,692 | 4,859 | 7,692 | 7,169 | 6,553 |
| HC2 | 10,988 | 4009 | 10,988 | 7,872 | 8,026 |
| HC3 | 4,797 | 6906 | 4,797 | 4,285 | 3,580 |
| HC4 | 1,838 | 17,566 | 1,838 | 1,516 | 1,253 |
| HC5 | 9,439 | 3,242 | 9,439 | 5,767 | 7,784 |
| N1b | 807 | 52,359 | 807 | 584 | 610 |
| N1a | 1,132 | 31,178 | 1,132 | 812 | 841 |
| N2b | 619 | 62,694 | 619 | 304 | 327 |
| N2a | 4,275 | 8,071 | 4,275 | 2,079 | 2,671 |
| N3b | 433 | 83,452 | 433 | 313 | 276 |
| N3a | 3,217 | 11,391 | 3,217 | 2,208 | 2,399 |
| N4b | 4,810 | 7,355 | 4,810 | 3,973 | 3,927 |
| N4a | 3,363 | 10,611 | 3,363 | 2,943 | 2,586 |
| N5b | 5,580 | 6,224 | 5,580 | 3,930 | 4,672 |
| N5a | 4,945 | 8,713 | 4,945 | 4,420 | 3,583 |
| N6b | 1,432 | 29,030 | 1,432 | 1,025 | 791 |
| N6a | 4,425 | 8,854 | 4,425 | 3,327 | 3,325 |

b, before; a, after;
